# The 'permeome' of the malaria parasite: an overview of the membrane transport proteins of *Plasmodium falciparum*

**DOI:** 10.1186/gb-2005-6-3-r26

**Published:** 2005-03-02

**Authors:** Rowena E Martin, Roselani I Henry, Janice L Abbey, John D Clements, Kiaran Kirk

**Affiliations:** 1 School of Biochemistry and Molecular Biology, Faculty of Science, The Australian National University, Canberra, ACT 0200, Australia; 2 Division of Neuroscience, The John Curtin School of Medical Research, The Australian National University, Canberra, ACT 0200, Australia

## Abstract

**Background:**

The uptake of nutrients, expulsion of metabolic wastes and maintenance of ion homeostasis by the intraerythrocytic malaria parasite is mediated by membrane transport proteins. Proteins of this type are also implicated in the phenomenon of antimalarial drug resistance. However, the initial annotation of the genome of the human malaria parasite *Plasmodium falciparum *identified only a limited number of transporters, and no channels. In this study we have used a combination of bioinformatic approaches to identify and attribute putative functions to transporters and channels encoded by the malaria parasite, as well as comparing expression patterns for a subset of these.

**Results:**

A computer program that searches a genome database on the basis of the hydropathy plots of the corresponding proteins was used to identify more than 100 transport proteins encoded by *P. falciparum*. These include all the transporters previously annotated as such, as well as a similar number of candidate transport proteins that had escaped detection. Detailed sequence analysis enabled the assignment of putative substrate specificities and/or transport mechanisms to all those putative transport proteins previously without. The newly-identified transport proteins include candidate transporters for a range of organic and inorganic nutrients (including sugars, amino acids, nucleosides and vitamins), and several putative ion channels. The stage-dependent expression of RNAs for 34 candidate transport proteins of particular interest are compared.

**Conclusion:**

The malaria parasite possesses substantially more membrane transport proteins than was originally thought, and the analyses presented here provide a range of novel insights into the physiology of this important human pathogen.

## Abstract 2

Bioinformatic and expression analyses attribute putative functions to transporters and channels encoded by the *Plasmodium falciparum* genome. The malaria parasite has substantially more membrane transport proteins than previously thought.

## Background

The malaria parasite (genus *Plasmodium*) is a unicellular eukaryote which, in the course of its complex life cycle, invades the erythrocytes of its vertebrate host. It is this intraerythrocytic phase of the parasite life cycle that gives rise to all the symptoms of malaria, a disease that is estimated to give rise to almost 5 billion episodes of clinical disease and up to 3 million deaths annually [1]. *Plasmodium falciparum*, the most virulent of the malaria parasites that infect humans, has developed resistance to most of the antimalarial drugs currently available. There is an urgent need for the development of new antimalarial drug strategies, and for an improved understanding of the mechanisms that underpin the parasite's ability to develop resistance to antimalarials.

Membrane transport proteins are integral membrane proteins that mediate the translocation of molecules and ions across biological membranes. They serve a diverse range of important physiological roles, including the uptake of nutrients into cells, the removal of unwanted metabolic waste products and xenobiotics (including drugs), and the generation and maintenance of transmembrane electrochemical gradients. These proteins play a key role in the growth and replication of the parasite, as well as in the phenomenon of antimalarial drug resistance. But despite this, and despite the fact that membrane transport proteins have proven to be extremely effective drug targets in other systems [2], all but a few of the membrane transport proteins of the malaria parasite remain very poorly understood, and their potential as antimalarial drug targets remains largely unexplored [2].

The 'permeome' is a term used here to describe the total complement of proteins involved in membrane permeability in a given organism. It encompasses the full range of channels and transporters encoded in the genome. The original annotation of the *P. falciparum *genome, published at the end of 2002, identified "a very limited repertoire of membrane transporters, particularly for uptake of organic nutrients" and "no clear homologs of eukaryotic sodium, potassium or chloride ion channels" [3]. It is questionable, however, whether this reflects a genuine paucity of such proteins in this organism, or simply shortcomings in the annotation.

Despite ongoing improvements in automated gene annotation, it is widely accepted that the routines involved provide a first phase of annotation and that the attainment of a high-quality annotation requires the intervention of manual curation (reviewed in [4]). Errors that are difficult to avoid in automated systems for genome annotation include the incorrect prediction of intron/exon boundaries and the position of the start/stop codons, which can result in incomplete or truncated proteins, or the merging of neighboring proteins [5,6]. The assignment of functional annotations to proteins is hampered by several factors [7], including the non-critical use of annotations from existing database entries, ignoring multidomain organization of the query proteins and/or the database hits, and, in the case of *P. falciparum*, the considerable divergence that generally exists between the parasite and those organisms for which sequence data is currently available in the databases [3]. Manual curation affords a greater flexibility in handling these problems.

For many of the predicted proteins encoded by *P. falciparum *the similarity to their closest non-*Plasmodium *homologs is insufficient to permit annotation on the basis of BLAST searches alone. As highlighted in a recent review of the current status of the malaria parasite genome project [8], the annotation of these proteins requires an in-depth assessment by a manual curator using a range of bioinformatic approaches [7] including position-specific iterated BLAST (PSI-BLAST), detection of conserved domains, construction of multiple sequence alignments and comparisons of predicted secondary structure. This process is laborious and time-consuming, but by combining the information gained from these analyses, it is possible to arrive at reliable annotations and to gain significant insight into the function of the proteins of interest.

In this paper we report the results of a detailed analysis of the permeome of *P. falciparum*. The study makes use of a computer program that searches a genome database on the basis of the hydropathy plots of the corresponding proteins [9]. The approach is based on the observation that the polypeptides comprising transporter proteins typically possess multiple hydrophobic transmembrane domains (TMDs) and connecting hydrophilic, extra-membrane loops that are detected as peaks and troughs, respectively, in a plot of the hydrophobicity index of the polypeptide. Many transporters characterized to date have between eight and 14 TMDs [10]. In searching for additional candidate transporters, the *P. falciparum *genome was therefore scanned for proteins with seven or more TMDs. Proteins retrieved by this search were subjected to a detailed analysis, involving the application of several different bioinformatic methods.

The analysis presented here has doubled the number of candidate membrane transport proteins identified in the genome, as well as attributing putative substrate specificities and/or transport mechanisms to all of those "transporter, putative" proteins previously lacking this information. The newly designated proteins include candidate transporters for nutrients such as sugars, amino acids, nucleosides and vitamins. There are also transport proteins predicted to be involved in maintaining the ionic composition of the cell and in the extrusion of metabolic wastes such as lactate. For 34 of the candidate transport proteins of particular interest we have investigated the time-course of expression of mRNA throughout the asexual blood stage of the parasite.

The enrichment in the repertoire of *P. falciparum*-encoded transport proteins reported here indicates that the parasite's permeome is not as impoverished as was originally thought.

## Results

### Parasite proteins with seven or more putative TMDs

**Figure 1:**
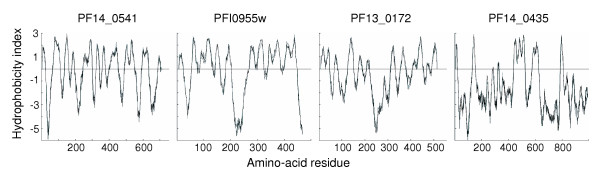
Comparison of the hydropathy plots of three *P. falciparum *proteins designated as putative transport proteins (PF14_0541, PFI0955w and PF13_0172) with that of a protein designated as having no putative function (PF14_0435). The PF14_0541 protein is a putative V-type H^+^-pumping pyrophosphatase (H^+^-PPase) and its hydropathy plot shows around 15 peaks in the hydrophobicity index, corresponding to 15 predicted transmembrane domains (TMDs) - as is characteristic of H^+^-PPases. The PFI0955w protein is a putative sugar transporter of the major facilitator superfamily (MFS) and its hydropathy plot indicates the presence of 12 TMDs. The PF13_0172 protein bears no sequence similarities with any known or putative transport proteins but its hydropathy plot shows around 11 peaks in the hydrophobicity index and resembles that of a typical transporter (for example, PF14_0541 or PFI0955w). The PF14_0435 protein has no non-*Plasmodium *sequence homologs or similarities to conserved domains, and although it is predicted to possess eight or nine putative TMDs, the hydropathy plot of the PF14_0435 protein does not resemble that of a typical transporter. The predicted TMDs are irregularly spaced (those in typical transporters tend to show more regularity of spacing, as in the first three hydropathy plots shown) and there are several very large extramembrane domains interspersed among the TMDs (many transporters have a single large extramembrane domain in the middle of the protein, but it is unusual for there to be multiple, irregularly spaced extramembrane domains of the type evident in PF14_0435). The possibility that the PF14_0435 protein (and others like it) is a transporter can certainly not be excluded; however there is simply not sufficient evidence to warrant its classification as such in the present study. The hydropathy plots were generated using the TMpred server [114].

A comprehensive search of the *P. falciparum *genome for genes encoding proteins predicted, on the basis of a hydropathy plot analysis, to have seven or more putative TMDs retrieved 167 candidate proteins. These proteins were categorized into three broad classes according to their putative functions (transport, non-transport or no putative function) as predicted by bioinformatic analyses. Known or putative transport functions were assigned to 89 (53%) of the retrieved proteins. A further 50 (30%) proteins were categorized as having functions that are non-transport related; these included various transferases, receptors, and proteins involved in trafficking and secretion (such as protein translocases), many of which have escaped annotation. The remaining 28 (17%) proteins had no non-*Plasmodium *sequence homologs or similarities to conserved domains, and did not resemble transporters in structure (see Figure 1); they therefore could not be ascribed a putative function.

### The expanding inventory of *P. falciparum *transport proteins

Most of the *P. falciparum *putative transport proteins retrieved by the hydropathy plot analysis used here belong to known transport families and are described in Additional data file 1. They include new additions to the major facilitator superfamily, the drug/metabolite transporter superfamily, and the P-type ATPase superfamily, as well as many others. Several families not previously identified in the genome, such as the voltage-gated ion channel superfamily, the peptide-acetyl-coenzyme A transporter family, the zinc-iron permease family and the multi antimicrobial extrusion family, were also found to have *P. falciparum*-encoded members.

A number of proteins to which we have assigned a putative transport function bear no significant sequence similarity to any functionally characterized proteins (transporters or otherwise) in the current databases. However, they do have hydropathy plots that resemble those of known transport proteins, consistent with the hypothesis that they too are transporters. These proteins fall into two categories: proteins that are related to 'hypothetical proteins' from other organisms (Additional data file 2); and novel, *Plasmodium*-specific proteins (Additional data file 3).

Transport proteins possessing six or fewer TMDs were not retrieved by our search criteria and for the most part have been omitted from the table in Additional data file 1. A limited number of such candidate transport proteins were identified in the original genome annotation and are as follows: nine members of the mitochondrial carrier family; a cation diffusion facilitator; five members of the ATP-binding cassette (ABC) superfamily; a V-type ATPase; an aquaglyceroporin (PfAQP [11]); and an arsenite-antimonite (ArsAB) effluxer. Two subunits are required to form a functional ArsAB efflux pump - an ATP-hydrolyzing component (ArsA) and a channel-forming integral membrane protein (ArsB). To date, only the ArsA protein has been identified in the *P. falciparum *genome and the absence of a parasite ArsB homolog may indicate that either the ArsA protein does not function as part of an ArsAB efflux pump or, alternatively, the ArsB protein is present but remains to be discovered. Likewise, the parasite has genes encoding the α, β, δ, ε and γ subunits of the catalytic F_1 _complex of an F-type ATPase as well as the *c *subunit of the membrane-spanning F_0 _component, but genes for the F_0 _*a *and *b *subunits have not yet been identified in the genome. Classification of the above proteins can be found at Ian Paulsen's TransportDB site [12]. The list of parasite transport proteins possessing six or fewer TMDs has recently been extended by the description of a *P. falciparum *homolog of an unusual bifunctional protein that contains an amino-terminal K^+ ^channel and a carboxy-terminal adenylate cyclase [13].

**Figure 2:**
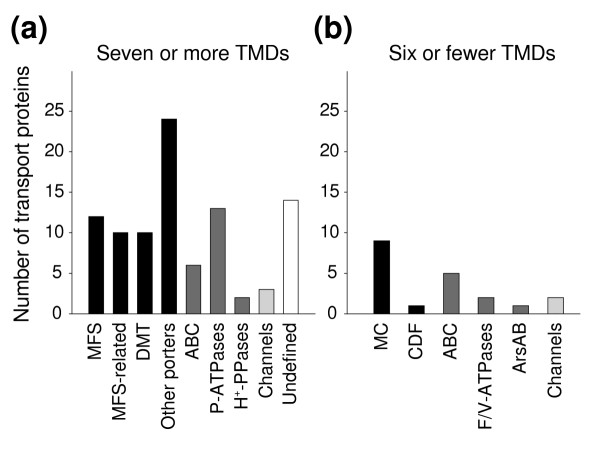
Graphical overview of the permeome of *P. falciparum*. **(a) **Transport proteins with seven or more transmembrane domains (TMDs). These proteins were retrieved by the analysis of the genome using a computer program that interrogates a genome database on the basis of the hydropathy plots of the corresponding proteins [9]. They include all the putative or known transport proteins with seven or more TMDs already identified in the genome, as well as 55 putative transport proteins with seven or more TMDs not previously recognized as such. **(b) **Transport proteins with six or fewer TMDs. These proteins were sourced in the most part from the annotated genome. Black bars, members of porter families (that is, uniporters, symporters and antiporters); dark-gray bars, members of primary active transporter families (that is, pumps); light-gray bars, members of channel families; white bars, putative transporters of unknown lineage and function. Abbreviations for the families are as follows: MFS, major facilitator superfamily; DMT, drug/metabolite transporter superfamily; ABC, ATP-binding cassette superfamily; P-ATPases, P-type ATPase superfamily; H^+^-PPases, H^+^-translocating pyrophosphatase family; MC, mitochondrial carrier family; CDF, cation diffusion facilitator family; F/V-ATPases, H^+^- or Na^+^-translocating F-type, V-type and A-type ATPase superfamily; ArsAB, arsenite-antimonite efflux family.

Only 54 transport proteins were identified in the original genome annotation and many of these are designated with generic descriptions such as 'transporter, putative', from which no information can be gained about the probable mechanism of transport or substrate specificity. Our analysis has retrieved a further 55 putative transport proteins, as well as attributing putative substrate specificities and/or transport mechanisms to all of those previously without (see Additional data file 1). This brings the total number of putative/proven *P. falciparum*-encoded transport proteins to 109. Of these, 61 are 'porters' (that is, uniporters, antiporters or symporters [10]), 29 are primary active transporters (that is, they utilize biochemical energy to pump solutes against an electrochemical gradient), five are channels, and 14 are putative novel transport proteins of unknown classification. Candidate transport proteins with seven or more TMDs (which were the subject of our search criteria) are shown in Figure 2a, whereas those with six or fewer TMDs (in the most part sourced from the annotated genome) are shown in Figure 2b.

### Predicting the cellular localization of *P. falciparum *transport proteins

Many transport proteins are located at the surface of the parasite, where they mediate the flux of solutes across the plasma membrane. Other transport proteins are found in the membranes of intracellular compartments such as those of the apicoplast, mitochondrion, digestive vacuole and organelles of the secretory pathway. The likely destination(s) within the cell of a given transporter can often be inferred by signals present in its polypeptide sequence and/or by its close homology to a transport protein of a known cellular localization. For example, the signal peptide required for the targeting of nuclear-encoded proteins to the parasite's apicoplast has been elucidated [14] and several *P. falciparum *transport proteins contain this type of signal (see Additional data files 1, 2 and 3). These putative apicoplast transporters include the parasite homolog of the plant chloroplast phosphoenolpyruvate:P_i _antiporters (PFE1510c [3]) as well as a putative amino-acid transporter (PFL1515c), several ABC transporters (PFC0125w, PF11_0466 and PF13_0271), P-ATPases (PFE0805w and PF07_0115) and other putative transport proteins of unknown function (PFL2410w, PF13_0172 and PFE1525w). Likewise, the nine parasite mitochondrial carriers contain putative signals for targeting these transporters to the mitochondrion. One of these, the putative phosphate carrier protein (MPC, PFL0110c), has been cloned and shown experimentally to possess mitochondrial targeting signals [15].

Two putative transporters involved in chloroquine resistance - the P-glycoprotein homolog 1 (Pgh1 [16]) and the 'chloroquine resistance transporter' (PfCRT [17]) - are localized to the parasite's digestive vacuole. PfCRT has recently been shown to be a member of the drug/metabolite transporter superfamily [18-20] and possesses several putative endosomal-lysosomal targeting signals (R.E.M. and K.K., unpublished work). The parasite V-type H^+^-ATPase is also found at the digestive vacuole membrane [21] and plays the major role in the acidification of the lumen [22]. There is experimental evidence for the presence of another proton pump at the vacuolar membrane, a K^+^-dependent, H^+^-translocating pyrophosphatase (H^+^-PPase) [22], although it is unclear which of the two parasite-encoded H^+^-PPases [23] is responsible for this activity. From its strong homology to Niemann-Pick type-C proteins (implicated in the efflux of lipids and cholesterol from lysosomes [24,25]) the PFA0375c protein is predicted to mediate the H^+^-coupled extrusion of lipids/sterols from the digestive vacuole. Likewise, the PFE1185w protein is predicted to reside at the digestive vacuole, based on its close homology to the endosomal Fe^2+ ^'NRAMP2' transporters (involved in the transferrin cycle [26]), and most probably catalyzes the H^+^-driven efflux of Fe^2+ ^into the cytoplasm.

Several transport proteins are dedicated to performing specialized tasks in the secretory pathway and specific 'retention' motifs participate in the sorting of these proteins between the membranes of the endoplasmic reticulum (ER) and the various Golgi compartments [27,28]. The nucleoside-sugar transporters are found exclusively at the membranes of the ER and Golgi apparatus of eukaryotes, where they mediate the uptake of nucleotide derivates (for example, UDP-galactose, UDP-glucose and GDP-fucose) from the cytosol in exchange for the corresponding nucleoside monophosphate (reviewed in [29,30]). The nucleotide sugars are then used by specific glycosyl-transferases to add sugar moieties to (glycosylate) proteins and lipids that are transported through the secretory pathway. The parasite's UDP-galactose:UMP antiporter homolog (which contains a retention motif) and other putative nucleotide-sugar transporters (such as PFB0535w and PFE0260w) are predicted to be residents of the secretory pathway organelles.

In the absence of any targeting signals or sorting motifs, membrane proteins are usually destined to follow the 'default' pathway and travel through the secretory pathway to the plasma membrane [31].

### Misannotation of transport proteins

Gardner *et al*. [3] inappropriately assigned a putative transport function to several *P. falciparum *proteins. The protein encoded by locus PFL0620c is designated as a putative choline transporter, yet it shares strong sequence similarities with known and putative glycerol-3-phosphate acyltransferases from a range of organisms, including the SCT1 protein of *Saccharomyces cerevisiae*. Indeed, the PFL0620c protein has recently been shown experimentally to be a glycerol-3-phosphate acyltransferase [32]. The annotation of PFL0620c as a transporter mostly probably arose from a misinterpretation of the function of SCT1 (Suppressor of a Choline Transport Mutant). As the name implies, the SCT1 protein was first identified in yeast for its ability to complement a growth defect caused by a deficiency in choline transport [33]. SCT1 was subsequently found to catalyze the acylation of glycerol 3-phosphate in the first step of phospholipid biosynthesis; hence, SCT1 restored growth in the mutant by stimulating the synthesis of phosphatidylcholine, not by increasing choline uptake [34].

The proteins encoded by the genes PF08_0098, PF11_0225, PF14_0133 and PF14_0321 are all annotated as putative ABC transporters, but none of these proteins contains more than a single putative TMD. Bioinformatic analyses indicate that the PF11_0225, PF14_0133 and PF14_0321 polypeptides are putative soluble ATP-binding proteins. PF11_0225 encodes a homolog of the *S. cerevisiae *GCN20 ATPase, which functions in association with the GCN1 protein to activate the translation initiation factor-2-alpha kinase (GCN2) in amino-acid-deprived cells [35]. The *Plasmodium *GCN20 ATPase has been cloned [36] and shown to complement the function of the yeast GCN20 ATPase by participating in the yeast translation regulatory pathway [37]. The PF14_0133 protein bears strong sequence similarities to the SufC proteins found in archaea, bacteria, cryptomonads, diatoms, dinoflagellates, red algae and plants. SufC is thought to be a versatile ATPase subunit that can interact either with the Suf(ABDSE) proteins to form a cytosolic complex for the assembly of Fe-S cluster-containing proteins, or with (unknown) membrane proteins to form an Fe-S ABC exporter [38]. PF14_0321 encodes for a short polypeptide (171 residues) which displays a weak homology to other soluble ATPases of unknown function from a wide range of organisms. Finally, the PF08_0098 protein is a member of the ABC1 family, which is distinct from, and unrelated to, the ATP-binding proteins of the ABC superfamily. ABC1 proteins are novel chaperonins essential for electron transfer in the *bc*_1 _segment of the respiratory chain (*S. cerevisiae *ABC1 [39]) and for ubiquinone production (*Escherichia coli *AarF [40]).

Gardner *et al. *[3] reported the presence of 16 P-type ATPases (P-ATPases) in the *P. falciparum *genome, although only 15 are listed at TransportDB [41]. Four of these - PFI1205c, PF10_0096, PF13_0137 and MAL13P1.352 - have no sequence similarities to known or putative P-ATPases, or to conserved domains of the P-ATPase superfamily. Furthermore, PF10_0096, PF13_0137 and MAL13P1.352 do not possess any putative TMDs. The PF13_0137 and MAL13P1.352 proteins display weak sequence similarities to conserved domains of the asparagine synthase (AsnB) and the nuclear cap-binding protein families, respectively, whereas the PF10_0096 protein is unrelated to any proteins or conserved domains in the current databases. PFI1205c encodes a large protein (1,249 residues) possessing 12-13 putative TMDs, and while this protein also lacks any similarities to conserved domains, it does appear to be a member of a putative transporter family specific to apicomplexans (see Additional data file 2).

### Expression of *P. falciparum *transport protein genes

**Figure 3:**
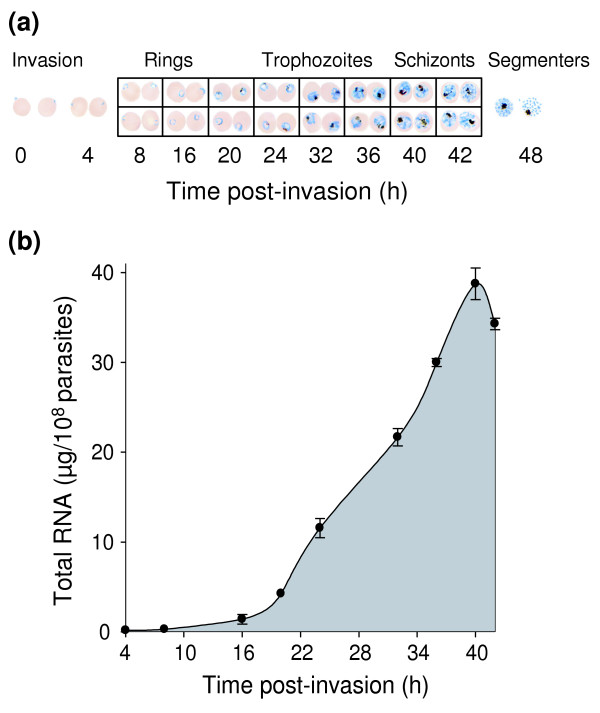
RNA obtained at different stages of *P. falciparum *development in the erythrocyte. **(a) **Representative Giemsa-stained *P. falciparum*-infected erythrocytes at the growth stages analyzed in this study. Samples from a tightly synchronized *P. falciparum *FAF6 culture were collected for the extraction of total RNA at ring (~4, 8, 16 and 20 h post-invasion), trophozoite (24, 32 and 36 h post-invasion) and schizont stages (40 and 42 h post-invasion). The cells depicted show the morphology of the parasitized cells in the culture at the given time point. The amount of RNA yielded from parasite cultures at around 4 h post-invasion was too low to warrant the inclusion of this time point in the subsequent gene-expression studies. Cells in the top row of boxes are from the first time course; cells in the bottom row are from the repeat time course (performed approximately 4 months later). **(b) **The quantity of total RNA inside the parasitized cell increases dramatically over the intraerythrocytic cycle. Total RNA was extracted from tightly synchronized *P. falciparum *FAF6 culture samples collected at nine stages (see above) over a single 48-h growth cycle of the intraerythrocytic parasite. The data are averaged from two different time courses performed approximately 4 months apart and are shown ± range/2.

The expression of 34 putative transport genes was analyzed throughout the asexual blood stage of the parasite. In previous studies, comparisons between the levels of transcripts present at different developmental stages of the parasite have been made from samples standardized to total RNA (see, for example [42-44]). In this study we quantified the amount of total RNA produced by the parasite as it progressed through the intraerythrocytic life cycle. As shown in Figure 3, the quantity of RNA in the infected erythrocyte increased significantly as the parasite grew from ring to trophozoite stage. There was 136 ± 19 (*n *= 2; ± range/2) times more total RNA in late trophozoites/schizonts (around 40 hours old) than in ring-stage parasites (around 8 hours old) and 161 ± 21 (*n *= 2; ± range/2) times more than in young rings (around 4 hours old). We therefore measured and compared transcript levels at different growth stages of the parasite from samples standardized to cell number rather than to total RNA (see below for further discussion).

In the following sections we consider in turn a number of different families of transport proteins, members of which have been identified and their stage-dependent mRNA expression characterized in this study.

### Members of the major facilitator superfamily

The major facilitator superfamily (MFS) is one of the largest classes of transporters; its members are prevalent in organisms from all kingdoms of life and are diverse in both sequence and function [45]. MFS transporters of the same subfamily tend to transport related substrates, and solutes transported by MFS proteins include sugars, metabolites, amino acids, peptides, nucleosides, polyols, drugs and organic and inorganic anions. The mechanism of transport also varies within the superfamily (and sometimes even within a subfamily) with examples of uniport, solute:solute exchange, solute:H^+ ^antiport, as well as Na^+ ^or H^+^:solute symport. Our analysis has doubled the parasite's complement of MFS transporters from six to 12, and while this still compares very poorly with other eukaryotes such as *S. cerevisiae *(85 MFS proteins) and *Caenorhabditis elegans *(137 MFS proteins), it surpasses that found to date in the parasitic eukaryote *Encephalitozoon cuniculi *(two MFS proteins) [41]. The *P. falciparum *proteins fall within either the sugar porter, drug:H^+ ^antiporter-1, monocarboxylate porter or peptide-acetyl-coenzyme A transporter families, although one protein displays only a weak relationship to the MFS and could not be placed reliably within a family (PFL0170w, see Additional data file 4).

**Figure 4:**
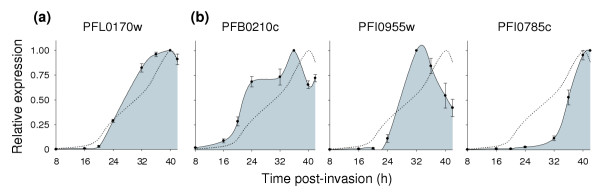
Stage-dependent gene expression of transporters throughout the intraerythrocytic cycle of *P. falciparum*. **(a) **A putative transporter of the MFS family; **(b) **the three *P. falciparum *members of the sugar porter family (a subfamily of the MFS). The PFB0210c gene encodes the *P. falciparum *hexose transporter, PfHT1 [46]. RT-PCR was conducted to semi-quantify the level of gene expression in ~1.5 × 10^4 ^parasitized cells at each growth stage. Relative expression (*y*-axis) is the ratio of the density of the band from the PCR product at each time point in the life cycle relative to that at the time point giving the largest yield of PCR product. Ratios calculated from replicate gels from the same PCR were averaged before the data from the two time courses (carried out approximately 4 months apart and each consisting of ≥ 2 PCRs) were combined to give the mean ± S.E. For comparison, the relative amount of total RNA in the parasitized cell over the same growth stages is also presented (dotted line).

The *P. falciparum *members of the sugar porter family include the hexose transporter (PfHT1/PFB0210c [46]), and the putative transporters PFI0785c and PFI0955w. Of these proteins, PfHT1 (which functions primarily to transport glucose) shows the greatest similarity to glucose transporters from other organisms, including mammals (Additional data file 4). In our expression analysis PfHT1 transcript was found to be present relatively early in the intraerythrocytic life cycle (around 8 hours post-invasion, Figure 4) and to increase rapidly in abundance between 16 and 24 hours, after which the level of transcript stabilized temporarily before increasing again to reach a maximum at approximately 36 hours. There is significant sequence homology between PFI0955w and PfHT1 (Additional data file 4); nevertheless, PFI0955w has diverged somewhat from the glucose transporters and may therefore catalyze the transport of other sugars or sugar-related substances. The transcription of PFI0955w was found not to begin until the parasite had spent some 24 hours inside the host cell (Figure 4); the level of transcript then very rapidly reached a maximum at around 32 hours and steadily decreased thereafter. PFI0785c bears some similarity to both PfHT1 and PFI0955w, but shows a closer resemblance to two putative MFS transporters from *Cryptosporidium parvum *and a putative plastid hexose transporter from *Olea europaea *(Additional data file 4). The PFI0785c transcript was almost undetectable until very late in the cycle, with the greatest increase in transcript level occurring between 32 and 40 hours.

**Figure 5:**
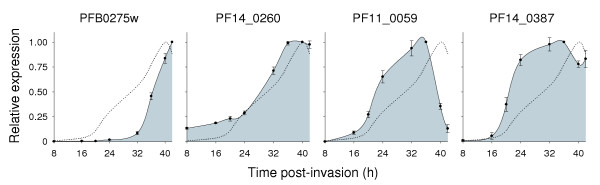
Stage-dependent gene expression of four putative members of the drug:H^+ ^antiporters-1 family (a subfamily of the MFS), throughout the intraerythrocytic cycle of *P. falciparum*. The analysis was carried out as described in the legend to Figure 4.

Five of the *P. falciparum*-encoded MFS transporters (the PFB0275w, PFE0825w, PF11_0059, PF14_0260 and PF14_0387 proteins) display a weak relationship with members of the 'drug-H^+ ^antiporter-1' family. PFB0275w and PF14_0260 share extensive amino-acid sequence homology with one another and are related to putative transporters from plants (see Additional data file 1 and 4). The expression profiles of these two genes were strikingly different: the PF14_0260 transcript was present at a low level very early in parasite development and reached a maximum over the 36-42-hour period, whereas transcription of PFB0275w occurred quite late in the cycle (Figure 5). The PF11_0059 protein is weakly related to putative multidrug resistance transporters but also bears some similarity to transporters of another subfamily of the MFS, the anion:cation symporter family (Additional data file 4). It is therefore possible that the PF11_0059 protein mediates the transport of organic anions, such as glucarate, biotin, phthalate or pantothenate (substrates of the anion:cation symporter family), rather than the efflux of drugs or metabolites such as polyamines, lactose or arabinose (substrates of the drug-H^+ ^antiporter-1 family). The level of PF11_0059 transcript increased rapidly between 16 and 24 hours and reached a maximum between 32 and 36 hours, after which it decreased dramatically (Figure 5). The closest BLASTP homolog of PFE0825w is a mouse protein designated as a 'putative organic cation transporter'. However, the mouse protein is not a member of the organic cation transporter family of the MFS, but does show good homology to a tumour suppressing STF-like protein from *C. elegans *and a weaker similarity to a putative tetracycline resistance protein from *Gloeobacter violaceus *(see Additional data file 4; sequences of several organic cation transporters are provided for comparison). The PF14_0387 protein also displays a weak similarity to the *G. violaceus *protein as well as to an *Escherichia coli *putative arabinose effluxer, and in the sequence alignment shown in Additional data file 4, the PFE0825w and PF14_0387 proteins are placed within the same cluster. The transcription of PF14_0387 increases rapidly between 16 and 24 hours, after which the level of transcript plateaued and then began to decrease after 36 hours (Figure 5). Expression of the PFE0825w gene was not studied.

**Figure 6:**
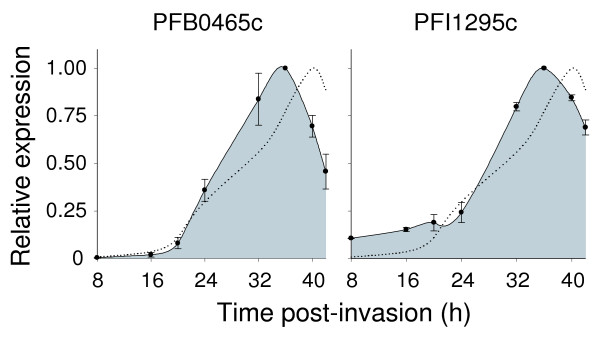
Stage-dependent gene expression of the two putative members of the monocarboxylate porter and oxalate:formate antiporter families (two closely related subfamilies of the MFS), throughout the intraerythrocytic cycle of *P. falciparum*. The analysis was carried out as described in the legend to Figure 4.

The PFB0465c and PFI1295c proteins share significant sequence similarities and are related to members of the monocarboxylate porter and oxalate:formate antiporter families. From the alignment shown in Additional data file 4, it appears that the PFB0465c protein resembles oxalate:formate antiporters, such as the OxlT-2 from *Archaeoglobus fulgidus*, whereas the PFI1295c protein is perhaps more similar to members of the monocarboxylate porter family such as the rat T-type amino-acid transporter and the human MCT-8 protein. The PFB0465c and PFI1295c genes had similar expression profiles (Figure 6); in both, the maximum level of transcript occurred at approximately 36 hours post-invasion. However, the transcription of PFI1295c began earlier in the development of the intraerythrocytic parasite.

The locus PF10_0360 appears to contain open reading frames (ORFs) for three different proteins. One of these (amino-acid residues 1,644-2,222) displays strong homology to the acetyl-CoA:CoA antiporters of the ER (Additional data file 4). Expression of the PF10_0360 gene was not studied.

### MFS-related families

**Figure 7:**
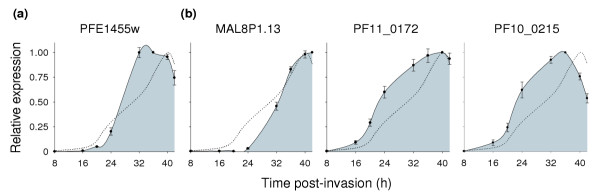
Stage-dependent gene expression of MFS-related transporters, throughout the intraerythrocytic cycle of *P. falciparum*. **(a) **A member of the glycoside-pentoside-hexuronide:cation symporter family; **(b) **the three *P. falciparum *members of the folate-biopterin transporter family. Both transporter families are distantly related to the MFS. The analysis was carried out as described in the legend to Figure 4.

The malaria parasite encodes members of the glycoside-pentoside-hexuronide:cation symporter (GPH), organo anion transporter (OAT) and folate-biopterin transporter (FBT) families, which are all relatives of the major facilitator superfamily [45]. The PFE1455w protein is a putative Na^+^- or H^+^-driven sugar symporter of the GPH family and the mRNA transcript of this gene was found to be most abundant between 32 and 40 hours post-invasion (Figure 7). The MAL6P1.283 protein belongs to a family of putative transporters from bacteria, plants and animals, members of which exhibit weak similarities to proteins and conserved domains of both the MFS and the OAT family (Additional data file 1 and 5). Members of the OAT family catalyze the transport of organic anion and cations and are found only within the animal kingdom. While the MAL6P1.383 protein and its relatives are only weakly similar to OAT proteins, in the absence of a more appropriate classification we have tentatively placed these proteins within the OAT family. Expression of the MAL6P1.383 gene was not studied.

The genes MAL8P1.13, PF11_0172 and PF10_0215 encode members of the FBT family. Proteins of this family are found only in cyanobacteria, protozoa and plants, and are thought to function as H^+ ^symporters. Thus far, only protozoan transporters have been characterized and these are known to mediate the uptake of the vitamins folate and/or biopterin (for example, FT1 [47] and BT1 [48] from *Leishmania *and FT1 from *Trypanosoma brucei *[49]). The MAL8P1.13 and PF11_0172 proteins share significant sequence similarities and as they are closely related to known or putative FBT proteins (see Additional data file 1 and 5), it is likely that they too catalyze the uptake of folate and/or biopterin. Both compounds contain the pteridine group, and members of this family may also transport pteridine (not itself a vitamin), though this has not been demonstrated directly. There is significant sequence divergence between the PF10_0215 protein and members of the FBT family and it is quite feasible that this protein transports other metabolites and/or vitamins. The expression profiles of the MAL8P1.13, PF11_0172 and PF10_0215 genes are compared in Figure 7.

### A family of novel putative transporters

**Figure 8:**
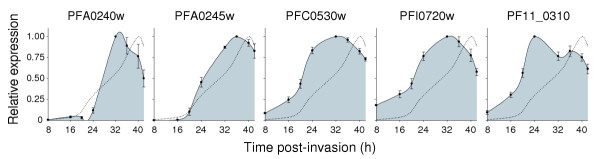
Stage-dependent gene expression of the five members of the novel putative transporter family, throughout the intraerythrocytic cycle of *P. falciparum*. The analysis was carried out as described in the legend to Figure 4.

We have assigned a putative transport function to 19 *P. falciparum *proteins that bear no significant sequence similarities to known or putative transport proteins, but which have hydropathy plots that are similar to those of known transporters. Within this group is a set of five proteins (PFA0240w, PFA0245w, PFC0530w, PFI0720w and PF11_0310) that share both sequence and structural homology, but which lack sequence similarity to any other proteins in the current databases. Several lines of evidence suggest that these proteins may share a common ancestry with transporters of the MFS, and for this reason they have been included in the table in Additional data file 1, where they are designated as *P. falciparum *novel putative transporters (PfNPTs). The PfNPTs share a common topology, consisting of 12 TMDs separated by a hydrophilic loop into two sets of six closely spaced TMDs (Additional data file 6). Such a topology closely resembles that found among transporters of the MFS and, consistent with this observation, one of the PfNPTs (PFA0245w) has a putative match to a conserved domain of the MFS. Furthermore, two or more iterations of a PSI-BLAST search of the National Center for Biotechnology Information (NCBI) database using a PfNPT as the query sequence retrieves, with good significance, several putative MFS proteins. A characteristic of most members of the MFS family is the presence of a conserved amino-acid sequence between TMDs 2 and 3 and a related but less conserved motif in the corresponding loop in the second half of the protein (between TMDs 8 and 9). As shown in Additional data file 6, each PfNPT protein contains a putative MFS-specific motif between TMDs 2 and 3 and between TMDs 8 and 9, consistent with the hypothesis that these proteins are distantly related to the MFS. The PFC0530w and PFI0720w genes were found to share a similar pattern of expression over the asexual blood stage of the parasite, whereas the remaining PfNPT genes exhibited quite different expression profiles (Figure 8).

### Amino-acid transporters

We have designated six *P. falciparum*-encoded proteins as putative amino-acid transporters. Three (MAL6P1.133, PFL0420w and PFL1515c) are members of the amino acid/auxin permease (AAAP) family. The other three (PFB0435c, PFE0775c and PF11_0334) are members of the neurotransmitter:Na^+ ^symporter (NSS) family. Proteins of the AAAP family are known to mediate the transport of a specific amino acid (for example, the proline permease of *Arabidopsis thaliana *[50]), or of a group of similar amino acids (for example, the neutral amino-acid permease of *Neurospora crassa *[51]), while several members exhibit very broad specificities, transporting all naturally occurring amino acids (for example, the general amino-acid transporter of *A. thaliana *[52]). AAAP proteins are found in yeast, protozoans, plants and animals, and transport is usually either H^+^- and/or Na^+^-dependent [53-55]. The MAL6P1.133 protein appears to share the greatest level of sequence similarity with amino-acid transporters from other protozoans, yeast and mammals (Additional data file 7). The PFL0420w and PFL1515c proteins are closely related (Additional data file 1) and appear to be most similar in sequence to amino-acid transporters from plants and insects (Additional data file 7). The PFL1515c protein contains a putative signal for targeting to the apicoplast membrane.

Substrates of NSS transporters include amino acids, neurotransmitters and other related nitrogenous compounds such as taurine (a sulfonic amino acid) and creatine. NSS proteins are found only in archaea, bacteria and animals, and most of the transporters characterized so far operate via a solute:Na^+ ^symport mechanism (for example, the tryptophan:Na^+ ^symporter of *Symbiobacterium thermophilum *[56] and the mammalian neutral amino acid:Na^+ ^symporter [57]). Most are also Cl^-^-dependent, for example the neutral and cationic amino acid: Na^+^:Cl^- ^symporter of humans [58]. Two exceptions are the absorptive amino-acid transporters - CAATCH1 [59] and KAAT1 [60] - from the gut epithelium of the insect *Manduca sexta*. These transporters catalyze the Na^+^-dependent (K_m _(Na^+^) ≈ 6 mM) or K^+^- dependent (K_m _(K^+^) ≈ 32 mM) transport of amino acids when expressed in *Xenopus *oocytes, but the low Na^+ ^(less than 5 mM) and high K^+ ^(aproximately 200 mM) concentrations prevalent in the insect gut lumen ensure that these transporters operate predominately via K^+ ^symport *in vivo *[60]. The *Plasmodium *NSS proteins, while retaining several of the conserved NSS sequence motifs, have diverged considerably from the other family members (Additional data file 1 and 8), making it difficult to ascertain a putative substrate(s) for each transporter. Nevertheless, it does appear that the parasite proteins may bear more similarities to the NSS members which transport amino acids, than they do to those which transport other neurotransmitters or osmolytes.

**Figure 9:**
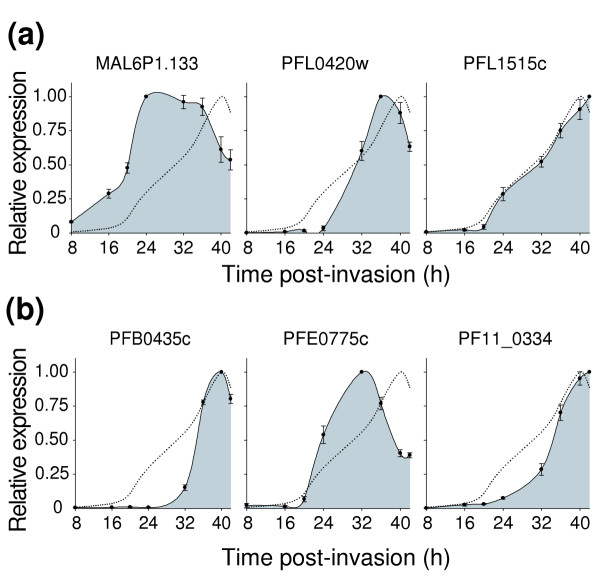
Stage-dependent gene expression of putative amino-acid transporters throughout the intraerythrocytic cycle of *P. falciparum*. **(a) **Amino acid/auxin permeases; **(b) **neurotransmitter:Na^+ ^symporters. The analysis was carried out as described in the legend to Figure 4.

Figure 9 shows the stage-dependent gene expression for each of the six *Plasmodium *putative amino-acid transporters. Significant levels of PFB0435c, PF11_0334 or PFL0420w transcript were present only in the second 24-hour period of parasite development and expression of the PFL1515c and PFE0775c genes began in earnest only slightly earlier (at around 20 hours). By contrast, there was a relatively high level of MAL6P1.133 transcript early in parasite development and the expression of this gene continued throughout the intraerythrocytic stage.

### The equilibrative nucleoside transporter family

**Figure 10:**
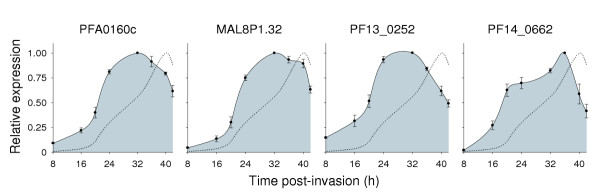
Stage-dependent gene expression of the four *P. falciparum *members of the equilibrative nucleoside transporter family, throughout the intraerythrocytic cycle of the parasite. The PF13_0252 gene encodes the *P. falciparum *nucleoside transporter, PfENT1 [62,63]. The analysis was carried out as described in the legend to Figure 4.

Members of the equilibrative nucleoside transporter (ENT) family mediate the uptake of nucleosides and/or nucleobases and are present in yeast, protozoa and animals. Transport via ENT proteins is not usually coupled to the movement of a driving ion (hence the name 'equilibrative'); the exceptions are three electrogenic nucleoside:H^+ ^symporters from *Leishmania donovani *[61]. A *P. falciparum*-encoded ENT, the PF13_0252 protein, has been characterized in *Xenopus *oocytes and shown to transport purine and pyrimidine nucleosides and nucleobases (PfENT1 [62,63]) and a second protein (MAL8P1.32) is annotated in the genome as a putative nucleoside transporter. We have identified two further *P. falciparum *putative nucleoside/nucleobase transporters, PFA0160c and PF14_0662. Each parasite ENT protein displays a predicted secondary structure that is characteristic of members of the ENT family - 11 TMDs with a large intracellular loop between domains 6 and 7. However, despite this conservation in structure, the four malaria proteins share limited sequence similarities with each other and are only very weakly related to ENT proteins from other organisms (Additional data file 1). The expression profiles of the PFA0160c, MAL8P1.32 and PF13_0252 genes were similar; in each there was a significant level of transcript present early in parasite development and a rapid increase in transcript abundance occurred between 16 and 24 hours, after which the level of transcript reached a maximum (at around 32 hours) and then declined slowly (Figure 10). By contrast, the PF14_0662 transcript increased in abundance rapidly between 8-20 hours and peaked at approximately 36 h.

### Inorganic anion transporters

**Figure 11:**
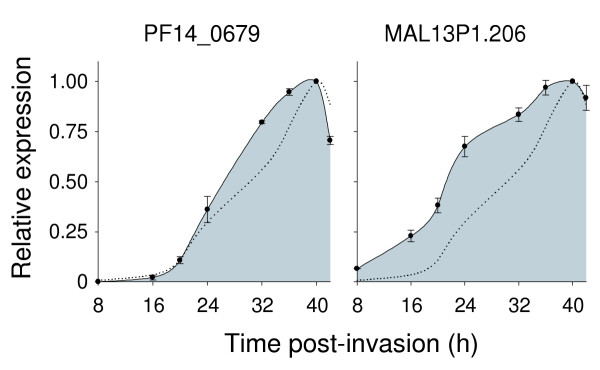
Stage-dependent gene expression of the putative inorganic anion exchanger (PF14_0679) of the sulphate permease family and the Pi :Na^+ ^symporter (MAL13P1.206) of the inorganic phosphate transporter family, throughout the intraerythrocytic cycle of *P. falciparum*. The analysis was carried out as described in the legend to Figure 4.

MAL13P1.206 and PF14_0679 are candidate inorganic anion transporters. The PF14_0679 protein bears strong sequence similarity to the bacterial members of the large and ubiquitous sulfate permease (SulP) family (Additional data file 1). None of the bacterial SulP proteins has been characterized functionally, but several of the plant members are known to be SO_4_^2-^:H^+ ^symporters and different mammalian SulP proteins carry out the following types of transport activities: SO_4_^2-^:HCO_3_^- ^antiport; HCO_3_^-^:Cl^- ^antiport; and the transport of SO_4_^2-^, formate, oxalate, Cl^- ^or HCO_3_^- ^in exchange for any one of these anions. As depicted in Figure 11, the level of PF14_0679 transcript is low in the first 16 hours of parasite development, but increased steadily thereafter, peaking at approximately 40 hours.

The MAL13P1.306 protein belongs to the family of inorganic phosphate transporters (PiT), members of which catalyze the Na^+^- or H^+^-dependent uptake of inorganic phosphate (P_i_). In the official annotation of the genome, the *P. falciparum *PiT protein (PfPiT) is designated as a putative P_i_:H^+ ^symporter. Yet in a BLASTP search of the NCBI database PfPiT retrieves the Na^+^-coupled P_i _transporters from animals and yeast with far greater significance than the H^+^-coupled P_i _transporters of bacteria and plant chloroplasts. This observation has been supported by a detailed phylogenetic analysis in which the *Plasmodium *PiT protein was found to cluster within the branch of Na^+^-dependent PiT proteins (R.E.M., K. Saliba, A. Bröer, C. McCarthy, M. Downie, R.I.H., R. Allen, S. Bröer and K.K., unpublished work). Subsequent flux experiments performed with trophozoite-stage parasites revealed the presence of a Na^+^-dependent P_i _transporter at the parasite plasma membrane, and the expression of the PfPiT protein in *Xenopus *oocytes has verified its function as P_i_:Na^+ ^symporter (R.E.M., K. Saliba, A. Bröer, C. McCarthy, M. Downie, R.I.H., R. Allen, S. Bröer and K.K., unpublished work). As shown in Figure 11, the PfPiT (MAL13P1.206) gene was expressed in the early stages of parasite development and the transcript became increasingly abundant after 16 hours, reaching a maximum at around 36-40 hours.

### The voltage-gated ion channel superfamily

**Figure 12:**
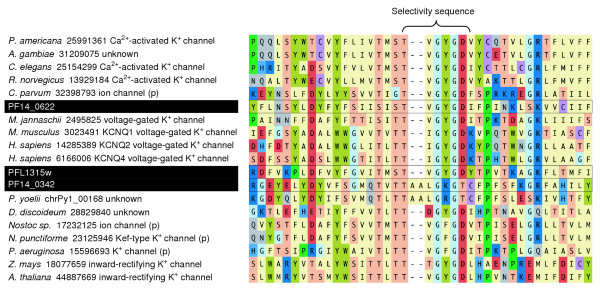
The alignment over the region of the selectivity sequence of the putative *P. falciparum *novel ion channel protein (PF14_0342) with a representative selection of K^+ ^channel proteins (known and putative) of the voltage-gated ion channel superfamily. *P. falciparum *sequences are boxed and the protein designators highlighted. The *P. yoelii *homolog of the PF14_0342 protein is encoded by the chryPy1_00168 locus, which is available at PlasmoDB. For proteins of other organisms, the NCBI accession number and the known or putative (p) function of the protein are given. A larger alignment of the voltage-gated ion channel superfamily, encompassing transmembrane domains (TMDs) 2-6, is presented in Additional data file 9. Residues are shaded as follows: blue, positively charged,; red, negatively charged; orange, hydoxyl; gray, amido; green, proline; purple, cysteine; mid-blue, histidine,; light blue, glycine; olive green, tryptophan and tyrosine; yellow, remaining nonpolar.

Members of the voltage-gated ion channel (VIC) superfamily are found in all domains of life. The channels characterized thus far are specific for K^+^, Na^+ ^or Ca^2+ ^under physiological conditions. Potassium channels of this superfamily are usually homotetrameric structures, assembled from a polypeptide subunit possessing six TMDs, and contain a central ion conduction pore (reviewed in [64,65]). Each subunit contains a highly conserved 'selectivity sequence' in the loop between TMDs 5 and 6, and in the tetrameric structure these loops are positioned together to form a 'selectivity filter' which determines the cation specificity of the channel. There is also a 'voltage sensor' in TMD 4, which consists of three to nine regularly spaced, positively charged amino-acid residues. There are three members of the K^+ ^channel family in the malaria genome (PFL1315w, PF14_0342 and PF14_0622), one of which has recently been cloned (PFL1315w [66]). Unlike most members of the family, the *P. falciparum *polypeptides are predicted to possess more than six TMDs; hence they were retrieved by our search criteria (which specified proteins with seven or more TMDs). The PFL1315w and PF14_0622 proteins display limited sequence similarities to known or putative K^+ ^channels from other organisms and are also only very weakly related to each other (see Additional data file 1 and 9), but both possess the signature selectivity sequence of the K^+ ^channel family (Figure 12 and Additional data file 9). A more extensive bioinformatic study of the PFL1315w and PF14_0622 proteins will be presented elsewhere (R. Allen and K.K., unpublished work).

The PF14_0342 protein is closely related to the PFL1315w protein and a sequence alignment of these two polypeptides reveals that this similarity extends over most of the lengths of the proteins (Additional data file 1 and 9). However, the PF14_0342 polypeptide has undergone some remarkable changes in the region of the ion-selectivity sequence. The most noteworthy of these are as follows: the insertion of two alanines, the presence of threonine in a position that, in almost every other member of the family, is occupied by aspartic acid, and the replacement of a neutral amino acid by a lysine two residues to the left of this position (Figure 12).

**Figure 13:**
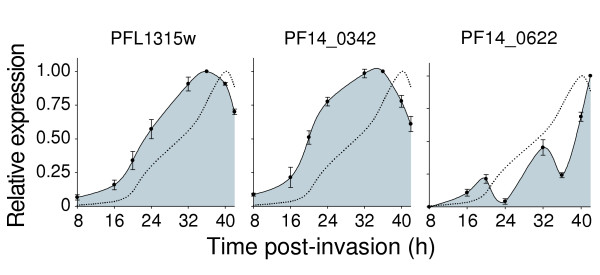
Stage-dependent gene expression of two *P. falciparum *putative K^+ ^channels (PFL1315w and PF14_0622) and the putative novel ion channel (PF14_0342) of the voltage-gated ion channel superfamily, throughout the intraerythrocytic cycle of *P. falciparum*. The analysis was carried out as described in the legend to Figure 4.

Figure 13 depicts the expression profiles of the PFL1315w, PF14_0342 and PF14_0622 genes. The PFL1315w and PF14_0342 transcripts were present in the early stages of parasite development and increased significantly in abundance between 16-24 hours, after which the level of transcript reached a maximum (at around 36 hours) and then declined. The PF14_0622 gene had a pattern of expression that was strikingly distinct from any other presented in this study; as the intraerythrocytic parasite matured the level of transcript appeared to rise and fall in successive waves of increasing amplitude.

## Discussion

### Enrichment of the *P. falciparum *permeome

In the original annotation of the *P. falciparum *genome, the parasite was described as possessing a very limited complement of transport proteins [3]. The detailed bioinformatic analysis presented here reveals that the parasite permeome is at least twice as large as first reported, and predicts the presence of a range of transport capabilities that were assumed previously to be lacking in the parasite. The newly designated proteins include candidate plasma membrane transporters for nutrients such as sugars, amino acids, nucleosides and vitamins. There are also transport proteins predicted to be involved in maintaining the ionic composition of the cell and in the extrusion of metabolic wastes such as lactate. Several of the new transporters are most probably located on intracellular membranes. Some of these are predicted to catalyze the flux of solutes either into or out of an intracellular compartment (for example, the putative iron effluxer of the digestive vacuole, PFE1185w), whereas others are predicted to mediate the exchange of metabolic intermediates between the cytosol and an organelle lumen (for example, the putative GDP-fucose:GMP antiporter of the Golgi, PFB0535w). A number of the *P. falciparum *proteins we retrieved with seven or more TMDs bear no significant sequence similarity to any other proteins (transporters or otherwise) characterized previously. Yet they have hydropathy plots that are similar to those of known transport proteins, consistent with the hypothesis that they too are transporters. Within this group is a subset of related proteins that form a novel family of putative transporters, which may be very distantly related to the MFS. These novel putative transporters appear to be specific to plasmodia and are therefore of potential interest as new antimalarial drug targets.

This enrichment in the repertoire of *P. falciparum*-encoded transport proteins indicates that the parasite permeome is not as impoverished as originally thought (although the parasite still cannot be considered to have a transporter-replete genome, see below). For instance, in the original study of the genome data it was suggested, on the basis of the apparent absence of an obvious amino-acid transporter, that the intraerythrocytic parasite must rely almost completely on the ingestion and digestion of host hemoglobin for its supply of amino acids [3]. However, the identification here of several putative amino-acid transporters, along with previous observations that the parasite is capable of both the import [67] and export [68] of amino acids, indicates that this is not the case. The six putative amino-acid transporters we identified display dissimilar mRNA expression patterns, suggesting that they fulfill different roles in the rapid development of the intraerythrocytic parasite. For example, the MAL6P1.133 protein is most closely related to amino-acid transporters from other protozoans, yeast and mammals, and the expression of this gene throughout the intraerythrocytic stage (Figure 9) suggests that the transporter has an important role in parasite growth, perhaps as a broad-specificity plasma membrane permease for amino acids. On the other hand, the PFL1515c protein is more similar to plant amino-acid transporters, the gene is expressed slightly later in parasite development (Figure 9), and the presence of a putative apicoplast targeting signal indicates that the protein probably mediates the transport of amino acids into and/or out of this organelle.

### *P. falciparum*-encoded channels

In their landmark paper Gardner *et al. *[3] reported "no clear homologs of eukaryotic sodium, potassium or chloride ion channels could be identified". However, two putative K^+ ^channels have since been cloned [13,66], and we have identified an additional putative K^+ ^channel (PF14_0622) as well as a novel protein of the K^+ ^channel family (PF14_0342). Our analysis of the *P. falciparum *genome did not reveal any putative Cl^- ^channels, and in this respect, our findings agree with the original annotation. Most organisms, including many other lower eukaryotes (for example, *Dictyostelium discoideum*, *Entamoeba histolytica *and various species of fungi), are known to encode at least one member of the ClC chloride channel family. ClC proteins possess 18 alpha helices [69], 10-12 of which are typically detected as putative TMDs by a TMD prediction program, yet our search criteria, which specified seven or more TMDs, did not retrieve a *P. falciparum*-encoded ClC protein. To verify this result, we carried out four iterations of a PSI-BLAST search of the NCBI database using the *E. coli *ClC protein (gi:26106498) as the query sequence. The search retrieved 695 ClC proteins from 226 different organisms, including many prokaryotes, unicellular eukaryotes, plants, and a broad range of animals, but a *Plasmodium *ClC homolog was not amongst the retrieved proteins. Also absent from the list of retrieved organisms were two other lower eukaryotes, the apicomplexan protozoan *C. parvum *and the microsporidian *E. cuniculi*, both of which have been reported as lacking a ClC protein [70,71]. *P. falciparum*, *C. parvum *and *E. cuniculi *are all obligate, intracellular parasites that reside in the cytoplasm of the host cell, and to date they are the only eukaryotes that appear to lack a ClC protein. It is tempting, therefore, to speculate that the loss of ClC proteins in these organisms is related to their parasitic, intracellular life style.

A primary role of plasma membrane Cl^- ^channels in non-excitable cells is in the volume-regulatory response to cell volume perturbation [72,73]. It is likely that within the relatively sheltered environment of the host cytoplasm, the parasite is not exposed to significant permutations in osmolarity and that it therefore does not require Cl^- ^channels for this purpose. Furthermore, Cl^- ^channels in the parasite plasma membrane would facilitate the distribution of Cl^- ^ions in accordance with the membrane potential. The membrane potential across the membrane of the mature trophozoite-stage parasite has been estimated as -95 mV [74]. The [Cl^-^] in the erythrocyte cytosol is of the order of 95 mM [75] and if Cl^- ^were allowed to distribute between the erythrocyte and parasite cytosols on the basis of the membrane potential the [Cl^-^] in the parasite cytoplasm would be of the order of 3 mM (calculated via the Nernst equation). On the basis of X-ray microanalysis data [76] it is likely that the [Cl^-^] in the parasite cytoplasm is an order of magnitude higher than this, consistent with Cl^- ^being actively accumulated by the parasite, rather than being allowed to equilibrate via Cl^- ^channels. The accumulation of Cl^- ^by the parasite might be mediated by either a Cl^-^:H^+ ^symporter (as is found in plants and fungi [77,78]) or perhaps a Cl^-^:anion exchanger (such as the protein we have annotated as a putative inorganic anion exchanger, PF14_0679).

The one role in which Cl^- ^channels have been implicated in the physiology of the intraerythrocytic parasite is in the formation of the 'new permeability pathways' (NPP) that mediate the increased traffic across the host erythrocyte membrane of a wide range of low molecular weight solutes, including polyols, amino acids, sugars, vitamins, and both organic and inorganic (monovalent) anions and cations [79]. The NPP show a marked preference for anions over cations, and for K^+ ^over Na^+ ^[80]. Their transport properties are those expected of anion-selective channels [81], and electrophysiological studies have confirmed the presence of such channels in the infected erythrocyte membrane [82-84]. It has been proposed that these channels are endogenous proteins, activated by stresses or stimuli associated with the parasite's invasion of the host cell [83,84]. However, the recent report of strain-specific variations in the properties of a novel inwardly rectifying anion-selective channel induced by the parasite in the host cell membrane is consistent with the hypothesis that this channel (termed PESAC for *Plasmodium *erythrocyte surface anion channel [85]) is, instead, parasite-encoded [86]. If this is the case, the lack of an obvious Cl^- ^channel in the *P. falciparum *genome indicates that it is likely to be a novel type of channel.

One protein that warrants further consideration in this context is PF14_0342, a very unusual member of the K^+ ^channel family; it is distinguished from all other K^+^-channel family proteins by the presence of several significant mutations in the region of the ion-selectivity sequence. These include the substitution of a highly conserved aspartic acid by threonine, the mutation of a neutral amino acid to a lysine two residues to the amino terminus of this position, and the insertion of two alanines (Figure 12). In K^+ ^channels, the conserved aspartic residue is located at the extracellular edge of the pore [64], where it may create an electrostatic field that 'funnels' K^+ ^ions into the channel opening. The replacement of this acidic residue by a neutral amino acid, combined with the appearance of a positively charged lysine residue nearby, raises the possibility that the ion selectivity of the PF14_0342 channel is no longer strictly cationic, and may even be anionic. The significance of the inserted alanines is difficult to predict, but they may serve to enlarge the diameter of the selectivity filter and thereby permit the transport of larger solutes. The putative ion channel PF14_0342 might therefore be considered as a candidate for the parasite-induced NPP, and the localization of the protein within the infected cell, and its physiological characteristics, are presently under investigation. If the PF14_0342 protein is indeed a component of the NPP, it may be anticipated that strain-specific differences in the electrophysiological characteristics of the parasitized erythrocyte [86] will correlate with a difference(s) in the amino-acid sequence of PF14_0342.

### Na^+^-dependent transporters and the physiological role of the NPP

Shortly after invasion by the malaria parasite, the concentration of Na^+ ^in the host erythrocyte cytosol is similar to that in uninfected erythrocytes, and to that in the cytosol of the parasite itself [76]. There is, therefore, little if any Na^+ ^concentration gradient across the parasite plasma membrane. With the induction of the NPP at around 12-15 hours, however, there is a progressive leakage of Na^+ ^into, and K^+ ^out of, the infected erythrocyte [80], resulting in a marked increase in the [Na^+^] in the erythrocyte cytosol and, therefore, a substantial inward Na^+ ^concentration gradient across the parasite's plasma membrane [76]. Our identification of several putative Na^+^-coupled transporters in the malaria parasite genome suggests that a significant component of the parasite's metabolism might depend on Na^+^-driven transport processes; the influx of Na^+ ^via the NPP, and the consequent increase in [Na^+^] in the infected erythrocyte cytosol may be important for this reason. The Na^+^-dependent P_i _transporter (PfPiT; R.E.M., K. Saliba, A. Bröer, C. McCarthy, M. Downie, R.IH., R. Allen, S. Bröer and K.K., unpublished work) provides one example of how this Na^+ ^gradient can be used to drive the accumulation of an essential nutrient. Other likely candidates for transporters able to utilize the Na^+ ^gradient across the parasite plasma membrane to energize solute transport include the three putative amino acid:Na^+ ^symporters of the NSS family, the putative Na^+^- or H^+^-driven sugar symporter of the GPH family, the putative MATE antiporter, and one or more of the *P. falciparum *MFS transporters.

### Standardization of gene-expression levels and stage-specific changes in the total RNA content of intraerythrocytic *P. falciparum*

The level of a target mRNA in a sample is usually standardized to an internal reference, such as the expression of a 'housekeeping gene', rRNA or total RNA, in order to compare the relative abundance of the transcript between different cell samples. Housekeeping genes, such as that for glyceraldehyde-3-phosphate dehydrogenase (GAPDH), were originally thought to be expressed at constant levels, regardless of cell type, developmental stage or experimental manipulation. However, it has become increasingly evident that the transcripts of housekeeping genes are not always maintained at constant levels in the cell [44,87,88]; nor is it likely that there are mRNA species which are. Likewise, the level of rRNA in the cell is also known to vary in relation to factors such as cell type and age [88,89]. Hence, the use of either a housekeeping gene mRNA or rRNA as an internal standard has lost merit [88], and standardization to total RNA has emerged as the method of choice for comparing mRNA levels between different cell samples [90,91].

Standardization to total RNA may be an appropriate strategy when the (average) amount of total RNA per cell is similar in each of the samples. It is less appropriate for quantifying levels of gene expression between cells of grossly differing transcriptional activities [88,90,91]. In cells that are very transcriptionally active (and hence contain a high total RNA content) a target transcript will appear to be at a disproportionately low level in comparison with that quantified in transcriptionally quiescent cells (containing less total RNA) in which the actual copy number of the target transcript is the same. In this study we measured the total RNA content of malaria parasites as they progressed through the intraerythrocytic lifecycle and found there to be around 160 times more RNA in late trophozoites/schizonts (around 40 hours old) than in young ring-stage parasites (around 4 hours old) (Figure 3). This is in agreement with previous findings of a considerably elevated rate of transcription in trophozoites compared with ring-stage parasites [92,93].

The substantial change in the RNA content of the parasite did not occur as a constant, linear increase from invasion through to maturity. Rather, the level of RNA remained very low early in the parasite's occupation of the erythrocyte (increasing only slightly in the first 16 hours), then increased rapidly between 20 to 40 hours and declined at 42 hours (Figure 3). This pattern correlates well with the onset at 20-30 hours of a broad range of metabolic activities in the parasite [92,94-96] and of the eventual downregulation of many of these activities in the late stages (schizont/segmenter) of parasite maturation [97-99].

Two recent large-scale studies have analyzed the transcriptome of the malaria parasite as it progresses through the intraerythrocytic cycle [42,43]. The Le Roch *et al. *[43] study examined the mRNA levels of approximately 95% of the predicted *P. falciparum *genes at six points in the intraerythrocytic cycle, whereas Bozdech and colleagues [42] measured gene expression at 1-hour intervals over the complete 48-hour cycle, thereby providing an impressive and comprehensive investigation of the transcriptome of asexual blood-stage *P. falciparum*. Both of these studies standardized mRNA levels to total RNA, which hinders gene-wise comparisons with the present work.

Nevertheless, it should be noted that the extent of similarity between the mRNA expression profiles obtained when standardizing to cell number (as in this study) and those obtained when standardizing to total RNA (as in the previous studies) depends to a large extent on the amount of transcript present in the ring-stage (the first 16-20 hours of the intraerythrocytic phase). For example, for those genes for which there is a substantial amount of transcript present in the ring stage, and which undergo a further increase during the second half of the intraerythrocytic cycle (for example, PFC0530w, PF11_0310 and MAL6P1.133), normalization of transcript level relative to total RNA will tend to show the mRNA level to be initially elevated relative to that seen in mature parasites and to then undergo a decrease as the parasite matures, whereas normalization to cell number will show a progressive increase in transcript level as the parasite matures (for example, Figures 8 and 9); that is, the two different types of profiles will look quite different. By contrast, for those genes for which transcript is rare or absent in ring-stage parasites but increases as the parasite matures (for example, PFB0435c, PFL0170w and PFA0245w), then the profiles obtained using the two different normalization methods are likely to share some resemblances, particularly in the second half of the intraerythrocytic cycle, where both modes of normalization will show increases in transcript levels. In summary, the relationship between the two datasets is highly complex, and the apparent differences or similarities observed between the profiles produced using these different methods will depend on such factors as the stage at which the transcription of a given gene begins, the rate at which the level of transcript increases, and the point at which the level of transcript reaches a maximum.

Standardization of mRNA levels to cell number, as in the present study, allows the level of a given transcript to be measured independently of the expression of other genes, rRNA or total RNA, all of which are parameters that are likely, or known, to change drastically during the intraerythrocytic cycle. The expression profiles we present reflect the number of copies of the transcript inside the cell and illustrate how this quantity changes as the parasite progresses from early ring stage through to schizont stages. From this it is immediately apparent how the expression of the gene is being regulated - independent of any other variable.

Standardization of transcript levels to cell number may also provide more insight into the likely biological role of the encoded protein, than standardization to RNA. This is best illustrated with a specific example. The parasite hexose transporter, PfHT1, provides the major route for the influx of glucose [46,100,101], an essential nutrient required by the intraerythrocytic malaria parasite for the production of ATP (via glycolysis). The rate of glycolysis is highly stage specific; in ring-stage parasites the level of glucose metabolism does not differ greatly from that measured in uninfected erythrocytes, but as the young parasite matures into a trophozoite there is a dramatic increase in the rate of glycolysis and the maximum level of glucose consumption occurs in the schizont stage [94,102,103]. Given the central role of PfHT1 in glucose uptake [46,100,101], it might be expected that the profile of *PfHT1 *expression will reflect the significant increase in the demand for glucose uptake by the maturing parasite. Indeed, when gene expression is standardized to cell number, the greatest increase in the level of PfHT1 mRNA occurs at the transition between the ring and trophozoite stages of the parasite (16-24 hours) and the maximum level is reached early in the schizont stage (Figure 4). Yet when standardized to total RNA (see [104]), the level of *PfHT1 *transcript appears to be most abundant in the ring-stage parasite (when relatively little glycolysis is occurring) and decreases rapidly as the parasite develops into a trophozoite, remaining at a low level throughout the parasite's most intense period of growth.

### Trends in the expression profiles of *P. falciparum *transport genes

All the genes for which mRNA expression was analyzed were transcriptionally active during the asexual intraerythrocytic stage of *P. falciparum*, albeit for varying durations. For 13 of the 34 genes investigated there was a readily detectable amount of transcript present from 8 hours through to 42 hours; these included genes encoding proteins known to transport inorganic phosphate (PfPiT), glucose (PfHT1) and nucleosides (PfENT1) and for putative transporters of nucleosides/nucleobases (PFA0160c and MAL8P1.32), amino acids (MAL6P1.133), monocarboxylates (PFI1295c) as well as a putative K^+ ^channel (PFL1315w) and novel ion channel (PF14_0342). Three of the 13 genes encode for members of the novel putative transporter family (PFC0530w, PFI0720w and PF11_0310) and while the likely substrate(s) of each of these transporters are unknown, the expression of the genes from early ring-stage parasites through to late schizonts suggests that these proteins each play an important role in the biochemistry of the intraerythrocytic parasite, presumably by mediating the transport of an important solute(s). By 16 hours post-invasion, the number of genes for which transcript could be detected had increased from 13 to 25, although in many cases the level of mRNA was still quite low (for example, the putative anion exchanger (PF14_0679) and three amino-acid transporters (PFE0775c, PF11_0334 and PFL1515c) amongst others). The transcripts of a few genes (PF14_0387, PF14_0342, PF14_0662 and PFL1315w) underwent a rapid increase in abundance between 16 and 20 hours, but for the majority of transport genes the rate of increase in transcript level was highest either between 20 to 24 hours (17 genes) or 24 to 32 hours (seven genes).

There is a small subset of transport genes which have expression profiles that depart from this trend. Transcripts of three transport genes (PFB0275w, PFB0435c and PFI0785c) were not detected until very late in the intraerythrocytic cycle (at 32 hours) and reached maximum abundance at 40-42 hours. These genes encode for putative transporters of drugs and metabolites, amino acids and sugars, respectively, and their selective expression in the late trophozoite and schizont stages suggests that the proteins are required for the development of late schizonts and/or merozoite morphogenesis. Perhaps the most intriguing pattern of gene expression presented in this study is that of the putative K^+ ^channel PF14_0622. Most transport genes in this study display a monophasic expression profile, with a single maximum and a single minimum, indicating that the gene is activated for transcription only once during the intraerythrocytic stage. This has been reported to be the case for the majority of *P. falciparum *genes expressed in the asexual blood cycle [42]. However, the expression of the PF14_0622 gene is strikingly distinct, in that the level of transcript rose and fell in successive waves of increasing amplitude as the parasite developed. The physiological significance of this expression pattern is unclear. Previous work has revealed that the timing of gene expression can determine the cellular localization of the resulting protein; when the apical membrane antigen-1 gene (*AMA-1*) is expressed in the late schizont/segmenter stage, the AMA-1 protein is targeted to the rhoptries (its normal destination), but the expression of *AMA-1 *in maturing trophozoites and during early schizogony (when rhoptries are absent) results in AMA-1 being targeted to the parasite plasma membrane as well as to a cytoplasmic location [105]. Therefore, one possibility could be that the PF14_0622 channel is being targeted to different membranes within the parasitized cell, depending upon the timing of expression.

### Comparison of the *P. falciparum *permeome with that of other organisms

The 54 transport proteins that were originally identified in the malaria genome account only for approximately 1% of the genes encoded by *P. falciparum*, and although our analysis has increased this to around 2.1% (109 proteins), this is still low, even when compared to other seemingly transporter-deficient microorganisms. For example, the archeon *Methanococcus jannaschii *is currently the prokaryote with the lowest percentage of transport proteins in its genome [106], but at 2.4% this is still higher than that found to date in *P. falciparum*. The intracellular parasite *E. cuniculi *has a remarkably reduced genome (around 2.9 Mb [71]) and encodes only 43 transport proteins [12]. However, these account for 2.2% of its genes; this unicellular eukaryote is therefore also slightly more transporter-rich than the malaria parasite. Other eukaryotic genomes have higher proportions of transport proteins; for example, *S. cerevisiae *(4.2%), *A. thaliana *(3.2%), *Drosophila melanogaster *(4.6%) and *Homo sapiens *(around 3.4%). At the high end of the spectrum is the *E. coli *genome, with an impressive repertoire of transporters accounting for 7.1% of the total number of genes [12].

The relative abundance of transport proteins can also be measured in terms of the number of transport genes per Mb of DNA. For the *P. falciparum *genome this is 4.7 (up from 2.3 estimated from the original annotation) transport genes per megabase, compared with *E. cuniculi *(17.2 per Mb), *S. cerevisiae *(22 per Mb) and an average of 36 transport genes per Mb across 18 species of prokaryotes [107]. The *Plasmodium *catalog of transport proteins is even more conspicuously meager when compared to the transporter-rich genomes of *E. coli*, *Bacillus subtilis *and *Hemophilus influenzae*, which have 66, 63 and 52 transport genes per Mb, respectively [12]. However, although the genomes of higher eukaryotes typically encode hundreds of transport proteins, they too have low numbers of transport genes relative to genome size (for example, *A. thaliana*, 6.7 per megabase and *D. melanogaster*, 5.3 per Mb). The human genome, which has the largest number of transport proteins (around 1,200), has the lowest relative abundance of transport genes to date (0.37 per Mb [12]).

The conclusion that the malaria parasite is 'minimalistic' with regard to transporters implies that there may well be relatively little redundancy (that is, the parasite tends not to have multiple transporters for particular roles). Compounds that inhibit a single transporter may therefore be highly effective as antimalarials, as the parasite is unlikely to have alternative transporters that it is able to use for the same purpose.

### Future work

Although the analysis reported here has increased significantly the number of transport proteins predicted to be present in *P. falciparum *it is highly likely that more parasite-encoded transport proteins remain to be uncovered. Our search criteria were targeted towards identifying transporters with seven or more TMDs; however, many of the polypeptides which form channels possess fewer than six TMDs and several types of transporters also contain fewer than six TMDs. The fact that the parasite has three putative channels (PFL1315w, PF14_0342 and PF14_0622) indicates that these types of transport proteins are indeed present in the *P. falciparum *genome. Applying the methodology used here to proteins which possess six or fewer TMDs is likely to identify additional transport proteins.

The identification of genes and the prediction of intron-exon structures in the *P. falciparum *genome are still being refined. For a number of the previously unannotated transport proteins there were inappropriate predictions for the 5'/3' ends and/or intron-exon boundaries (for example, PF14_0387, PFI1295c, PF10_0360, PF10_0215) and it is most likely this that led to their being overlooked in the original annotation process. Subsequent revisions of the genome data, using the latest gene-finding tools, comparative genomics and the integration of full-length cDNA sequences and proteomics data, will greatly improve the existing annotation of the genome and this, in turn, is likely to lead to further additions to the *P. falciparum *permeome.

## Materials and methods

### Identifying putative membrane transport proteins

A file containing the amino-acid sequences of all predicted proteins from the genome was obtained from the official *Plasmodium *genome database, PlasmoDB [108,109]. Polypeptides with seven or more TMDs were retrieved from this dataset using a computer program described previously [9]. Briefly, in an automated process, each polypeptide sequence was converted to a hydropathy plot and the number of peaks, corresponding to putative TMDs, detected. Proteins which satisfied the search criteria (those having between 250 and 5,000 residues in length and with seven or more TMDs; see Additional data file 10 for full details) were retrieved, and duplicate sequences were removed.

The TMD-based search tool at PlasmoDB was used to search for further putative membrane proteins. In these analyses, either the TMHMM2 or TMpred algorithms were used to scan for proteins possessing 7-25 TMDs. The list of proteins retrieved by the TMHMM2 and TMpred programs was compared with that already retrieved using the original program, and while there were a few proteins that the original program had retrieved and PlasmoDB had not, and *vice versa*, the results were mostly in agreement. The additional proteins identified at PlasmoDB were included in the subsequent annotation studies.

### Annotation of proteins

A combination of bioinformatics tools was used to assign putative functions to the (probable) membrane proteins retrieved from the hydropathy plot analysis. Each sequence was queried against the NCBI nonredundant protein database (using BLASTP [110]) and the Entrez Conserved Domain Database (using reverse position-specific BLAST [111]) in order to determine its relationship to other proteins. The values presented in Additional data file 1 are from analyses carried out in the second half of 2003. In some instances, there was only a weak similarity between the *P. falciparum *protein and a Conserved Domain Database entry and/or the closest (non-*Plasmodium yoelii*) BLASTP homolog. For such cases, the possibility of a common ancestry between the *Plasmodium *protein and proteins from other organisms was explored further by performing several iterations of a PSI-BLAST [112] search of the NCBI database. Comparisons of both protein sequence and predicted secondary structure (see below) were used to evaluate alternate models for each gene; in several cases it was found that a gene prediction other than the 'official' model provided a more likely candidate protein. Where applicable, *P. falciparum *proteins were placed into known transport protein families according to the official transporter classification system [10].

### Secondary structure predictions

Related transporters (comprising a protein family) show marked similarities in their predicted secondary structures, even when they share a relatively low level of sequence homology [113]. The hydropathy plot of each *P. falciparum *protein was compared with that of its BLAST homologs to investigate whether the observed sequence homologies corresponded to a similarity in the predicted structures of the proteins. Such an analysis is of particular assistance when the sequence similarity shared by two proteins is weak; a good agreement between their respective secondary structures adds support to the hypothesis that these proteins share a similar function. It also serves to identify those *P. falciparum *putative transporters that are truncated or fused to proteins from adjacent genes as a result of errors in the prediction of gene structures. The number and spatial arrangement of putative membrane-spanning domains in a polypeptide was predicted using TMpred [114] and TMMHM v2.0 [115].

### Construction of alignments

The ClustalW program [116] in MacVector 7.1 was used to generate and edit all alignments. Alignments were converted to PDF form and compiled in Adobe Photoshop 6.0.1. Full-length versions of the alignments presented or mentioned in this paper are available from the authors upon request.

### Preparation of *P. falciparum*-infected red blood cells

Human red blood cells (type O^+^) infected with *P. falciparum *(strain FAF6) were cultured as described previously [74] using a method adopted from Trager and Jensen [117]. The hematocrit was maintained at 4% and the parasitemia at 13-16%. Tightly synchronized cultures were achieved by repeated applications of sorbitol lysis [118] over a 9-day period immediately before the RNA extractions commenced. Cell counts were made using an improved Neubauer counting chamber and both the culture parasitemia and the parasite growth stage were assessed by methanol-fixed, Giemsa-stained blood smears. Photographs of the blood smears were taken with a SPOT RT colour camera connected to a Leica DML microscope and images of parasite-infected red blood cells were compiled in Adobe Photoshop 6.0.1.

### Isolation of total RNA

Culture samples were collected at nine stages over a single intraerythrocytic growth cycle of the parasite and total RNA was extracted using the NucleoSpin RNA II kit (Macherey-Nagel) in accordance with the 'high yield protocol' supplied by the manufacturer. This procedure is reported to recover 90-100% of the RNA bound to the column and in control experiments in which as little as 200 ng of RNA was loaded onto a column we confirmed this to be the case (data not shown). A NucleoSpin column can bind a maximum of 100 μg of nucleic acid and the manufacturers recommend that no more than 10^9 ^cells should be loaded per column; exceeding this quantity may cause clogging of the column, leading to poor RNA yields. However, approximately 85% of the cells in a parasite culture are mature human red blood cells, which lack a nucleus or other organelles and do not produce nucleic acids. We therefore investigated what quantities of parasite-infected red blood cell culture could be loaded onto a column without compromising the RNA yield. Initial experiments revealed that trophozoite-stage parasites contain considerably greater quantities of RNA and DNA than the less mature ring-stage parasites. We determined that for ring-stage parasites, increases in the cell sample from 9 × 10^6 ^(6 × 10^7 ^including uninfected red blood cells) to 9 × 10^7 ^(6 × 10^8^), and even up to 2.7 × 10^8 ^(1.8 × 10^9^), still gave proportionate increases in the RNA yield, indicating that the binding capacity of the column was not exceeded within this range. For mature trophozoites, however, the relationship between the cell sample size and the amount of RNA extracted became nonlinear when more than 6 × 10^7 ^(4 × 10^8^) cells were applied to the column. The quantities of parasites from which RNA was extracted (per column) are as follows: ring-stage, 1.3 × 10^8 ^parasites (8.7 × 10^8 ^cells in total including uninfected red blood cells); late ring stage/early trophozoite, 8.8 × 10^7 ^parasites (5.86 × 10^8 ^cells in total); and mature trophozoite/schizont, 4.4 × 10^7 ^parasites (2.93 × 10^8 ^cells in total). The amount of RNA extracted from the first time point (~4 h post-invasion) was exceedingly low (< 0.3 μg/10^8 ^parasites) and insufficient to warrant this sample's inclusion in the subsequent gene-expression analyses.

### Reverse transcription

cDNA was synthesized from 2 μg of total RNA (DNA-free) using SuperScript II RNase H^- ^Reverse Transcriptase (Invitrogen). First-strand synthesis was primed with Oligo-dT (Invitrogen) and the relative dNTP concentrations were adjusted to reflect the AT-bias of *P. falciparum *DNA (40% dATP, 40% dTTP, 10% dCTP, 10% dGTP). The disaccharide trehalose was added to the reaction (0.6 M) to improve the efficiency of reverse transcription. The action of trehalose is twofold: it stabilizes, and even activates, the reverse transcriptase protein at unusually high temperatures, and regions of secondary structure in the transcript (that otherwise cause the premature dissociation of the enzyme) are reduced or eliminated by both trehalose and the increased temperature of the reaction [119]. Reaction conditions were as follows: 42°C for 30 min, 60°C for 1.5 h, then 70°C for 15 min to inactivate the reverse transcriptase.

### PCR

Changes in mRNA levels throughout the asexual blood stage of the parasite of 34 proven/putative transport genes were semi-quantified by two-step RT-PCR using the principles developed by Halford and colleagues [120,121] and Fuster *et al. *[122]. As described above, the amount of total RNA in the cell increases significantly as the parasite matures (see Figure 3 for quantitative analysis). For this reason, for each time point the quantity of cDNA added to the PCR was standardized to cell number rather than to total RNA (see Discussion).

PCR was performed using the Platinum *Taq *PCRx DNA polymerase Kit (Invitrogen). Each reaction had a final volume of 50 μl and consisted of: 1x PCRx amplification buffer, 1x PCR enhancer solution, 1.5 mM MgSO_4_, dNTPs (320 μM dATP, 320 μM dTTP, 80 μM dCTP and 80 μM dGTP), 1.25 U Platinum *Taq *DNA Polymerase, 2 μM of each primer and 5 μl of template cDNA (at an appropriate dilution). Samples were amplified in a MJ Research PTC-200 Peltier thermal cycler and 20 μl of each reaction was loaded onto a 1% (w/v) agarose gel containing 0.5 μg ethidium bromide/ml. Electrophoresis was carried out at 70 V for exactly 90 min in a B2 Owl Separation System. On a separate gel, a duplicate aliquot of 20 μl from each reaction was loaded (in the reverse order) and electrophoresed in the same manner. PCR products were visualized with a UV transilluminator and the gels were photographed using a Gel Doc System camera linked to a computer running NIH Image software. Densitometric analysis of gel images was performed using ImageQuant V3.3 software (Molecular Dynamics).

Halford *et al. *[120] have shown that in a PCR where the concentration of template is limiting and the amplification of primer-dimers is occurring, the yield of product is dependent upon the logarithm of template cDNA input, even after 35 cycles of amplification. Furthermore, this relationship holds from the lowest concentration of template that gives a detectable yield, to amounts that are at least 100-fold (and up to 1,000-fold) greater than this concentration. A preliminary PCR revealed that the amount of product synthesized from trophozoite-stage cDNA varied between the 34 genes - a reflection, perhaps, of differences in transcript levels between these genes, differences in primer efficiency, or differences in the efficiency at which a given transcript species was reverse transcribed into cDNA. A subset of six genes, representative of this range in PCR product yield, was selected for inclusion in an experiment aimed at establishing a set of conditions under which, for each gene and cDNA sample, the final yield of PCR product was proportional to the amount of starting cDNA template. In brief, cDNA from ring (~20 h old), trophozoite (~32 h old) and schizont-stage (~42 h old) parasites was serially diluted and PCR product yields were measured after 15, 20, 25 or 30 cycles of amplification. It was found that using a moderately high dilution of cDNA (equivalent to around 3.8 × 10^4 ^parasites per 50 μl PCR), and amplification for 25 cycles, resulted in a proportional relationship between the concentration of input cDNA and the yield of PCR product for most genes and cDNA samples (data not shown). However, for a small subset of genes (PFA0245w, PFC0530w, PFE0775c, PFE1455w, PFI0720w, PF11_0334, PFL0420w, PF14_0622), these conditions did not result in quantities of product that could be measured reliably by densitometry and these genes required an additional five rounds of amplification before the yields could be measured reliably and the results were reproducible. Polymerase chain reactions were thermal cycled as follows: 94°C for 2 min, then 25 or 30 of 94°C for 30 sec, 55°C for 30 sec and 68°C for 45 sec.

The absence of contaminating DNA in the RNA samples was verified by the lack of PCR products formed in reactions (30 cycles) carried out for each primer set using RNA as the template (that is, 'minus RT' controls).

The extraction of RNA from parasites over the intraerythrocytic growth cycle was performed twice (4 months apart) and gene expression was analyzed within a time course. PCR product yields for a given gene are presented as a ratio of the signal measured for the specified time point, divided by that of the time point which gave the greatest yield of product. Ratios from replicate gels from the same PCR were averaged and the data from time course 1 and 2 (each consisting of at least *n *= 2 polymerase chain reactions) were combined to give the mean ± standard error. Each 50 μl PCR contained an amount of cDNA equivalent to around 3.8 × 10^4 ^parasites and product yields were measured from 2 × 20 μl aliquots of the reaction. Thus in this study, the relative level of transcript at each growth stage was estimated from around 1.5 × 10^4 ^parasitized cells.

### Primers

Oligonucleotide primers that would amplify a product of approximately 400 bp from each transcript of interest were designed using Primer3 [123]. These were synthesized by Invitrogen and for each primer pair, the gene identification, primer sequences, and product size are provided in Additional data file 10.

## Additional data files

The following additional data are available with the online version of this paper. Additional data file 1, 2 and 3 contain tables that summarize the known and putative transport proteins of *P. falciparum*. Additional data file 4, 5, 6, 7, 8 and 9 contain protein sequence alignments; Additional data file 10 contains supplementary methods.

## Supplementary Material

Additional File 1 Proteins that share sequence similarities with known or putative transport proteins and/or conserved domains of transport protein families Click here for file

Additional File 2 Proteins that are related to 'hypothetical proteins' from other organisms and have predicted secondary structures that resemble those of characterized transport proteins, but which do not share sequence similarities with known or putative transport proteins and/or conserved domains of transport protein families Click here for file

Additional File 3 *Plasmodium*-specific proteins that have the (predicted) structural characteristics of a transporter, but which, apart from strong sequence similarities to hypothetical proteins from other *Plasmodium *species, do not display any similarities to proteins or conserved domains in the current databases Click here for file

Additional File 4 The region over TMDs 1-5 of the alignment is shown. Sequences are separated into clusters of well-related sequences and are grouped as subfamilies of the MFS. *P. falciparum *sequences are boxed and the protein designators highlighted. For proteins of other organisms, the NCBI accession (gi) number and the known or putative (p) substrate specificity of the transporter are given. In some proteins, one or more of the extramembrane loop regions have been truncated and this is indicated by a solid black line. Residues are shaded as follows: positively charged, blue; negatively charged, red; hydoxyl, orange; amido, grey; proline, green; cysteine, purple; histidine, mid blue; glycine, light blue; tryptophan and tyrosine, olive green; remaining nonpolar, yellow Click here for file

Additional File 5 Both transporter families are distantly related to the MFS. The region over TMDs 2-5 and TMD 8 of the organo anion transporter family alignment is shown. The region over TMDs 3-5 and TMD 10 of the folate-biopterin transporter family alignment is shown. Legend as described for Additional data file 4 Click here for file

Additional File 6 (A) Hydropathy plots of two representatives of the novel putative transporter family: the PFI0720w and PFC0530w proteins. The two profiles are very similar and in each, there are 12 clear peaks in hydrophobicity, corresponding to 12 TMDs. A topology of 12 TMDs separated, by an extended extramembrane loop, into two sets of 6 closely spaced TMDs is characteristic of transporters of the MFS. (B) The alignment of the five *P. falciparum *novel putative transporters. The region over TMDs 2-3 and TMDs 8-9 of the alignment is shown. Members of the MFS family typically possess a conserved amino acid motif between TMDs 2 and 3 and a related but less conserved motif in the corresponding loop in the second half of the protein (between TMDs 8 and 9). The putative novel transport proteins also appear to contain these MFS-specific motifs between TMDs 2 and 3 and, to a lesser extent, between TMDs 8 and 9. For comparison, MFS-specific motifs from a range of known and putative MFS proteins are presented. Legend as described for Additional data file 4 Click here for file

Additional File 7 The region over TMDs 1-5, TMD 7 and TMD 10 of the alignment is shown. The sequences are separated into two clusters, one containing plant and insect proteins and the other protozoan, yeast and mammalian proteins. The PFL0420w and PFL1515c proteins appear to be more closely related to the former group of transporters, whereas the MAL6P1.133 is more similar to the latter. Legend as described for Additional data file 4 Click here for file

Additional File 8 The region over TMDs 1-3 and TMDs 6-10 of the alignment is shown. The sequences are separated into two clusters, one containing proteins known to transport neurotransmitters, creatine or taurine, and the other proteins known or hypothesized to transport amino acids. The *P. falciparum *proteins have diverged considerably from the other family members, but are perhaps most similar in sequence to the amino acid transporters. Legend as described for Additional data file 4 Click here for file

Additional File 9 The region over TMDs 2-6 of the alignment is shown. The locations of the 'voltage sensor' and 'selectivity sequence' are indicated. Legend as described for Additional data file 4 Click here for file

Additional File 10 Additional methods Click here for file
