# Supplementary material for: The 'permeome' of the malaria parasite: an overview of the membrane transport proteins of Plasmodium falciparum
Source: Genome Biol. 2005 Mar 2;6(3):R26. doi: 10.1186/gb-2005-6-3-r26 (PMC1088945; doi:10.1186/gb-2005-6-3-r26)
Supplement: Additional File 1 — Proteins that share sequence similarities with known or putative transport proteins and/or conserved domains of transport protein families [file gb-2005-6-3-r26-S1.pdf]

Table 1: *P. falciparum* putative transport proteins: proteins that share sequence similarities with known or putative transport proteins and/or conserved domains of transport protein families.

Gene Product[a]	Ch[b]	Annotation[c]	Size[d]	TMD[e]	Closest BLASTP Homologue[f]	E[g]	Conserved Domain Match[h]	Putative Function[i]
**Major Facilitator Superfamily** (TC 2.A.1)								
PFL0170w	12	Hypothetical protein	1250	12	Although PFL0170w does not have a sequence homologue in the current databases, it does display some structural similarity to the MFS proteins.	-	Pfam00083 0.028 (p)	Transporter
**Sugar Porter Family** (TC 2.A.1.1; Pfam00083)								
PFB0210c	2	Monosaccharide transporter, putative	504	12	***T. gondii* facilitative glucose transporter** (21630202; $1.7e^{-86}$ to Pfam00083).	$1.3e^{-105}$	Pfam00083 $7.5e^{-92}$	Hexose transporter 1 (PfHT1; [46])
PFI0955w	9	Sugar transporter, putative	476	11/12	**PFB0210c PfHT1** (23613713).	$3e^{-28}$	Pfam00083 $8e^{-12}$	Sugar transporter
PFI0785c	9	Transporter, putative	1379	11/13	***C. parvum* putative MFS protein** (46229527; $7e^{-05}$ to Pfam00083).	$4e^{-40}$	Pfam00083 $1e^{-05}$	Sugar transporter
**Drug:H^+ Antiporter-1 Family** (TC 2.A.1.2; COG2814)								
PFB0275w	2	Membrane transporter Hypothetical protein, conserved	565	11	***A. thaliana* putative transporter** (15234530; $1e^{-119}$ to KOG1330 and $6e^{-07}$ to COG2814).	$9e^{-59}$	KOG1330 $2e^{-25}$ COG2814 $25e^{-09}$	Metabolite/drug transporter
PF11_0059*	11	Hypothetical protein	417	9	***P. luminescens* putative multidrug resistance transporter** (36787367; $3e^{-14}$ to COG2814)	$9.9e^{-04}$	Pfam00083 0.002 (p)	Metabolite/drug transporter
PF14_0260	14	Hypothetical protein	809	12/11	**PFB0275w** (23593282).	$4.6e^{-22}$	COG2814 $8e^{-04}$	Metabolite/drug transporter
PF14_0387 FullPhat	14	Hypothetical protein	582	12	***G. violaceus* putative tetracycline resistance protein** (37521059; $8e^{-09}$ to COG2814).	$3e^{-07}$	COG2814 $1e^{-11}$	Metabolite/drug transporter
PFE0825w	5	Transporter, putative	440	10/11	***M. musculus* putative organic cation transporter** (6679180; $1e^{-05}$ to KOG2615).	$2.1e^{-23}$	KOG2615 0.003 (p)	Metabolite/drug transporter
**Monocarboxylate Porter Family** (TC 2.A.1.13; KOG2504, PfamB252, PfamB1302)/ **Oxalate:formate antiporter Family** (TC 2.A.1.11; KOG2504, PfamB252)								
PFB0465c	2	Membrane transporter Hypothetical protein	457	12/11	***C. briggsae* hypothetical protein** (39587811; $4e^{-33}$ to KOG2504, $3.5e^{-09}$ to PfamB252 and $4.7e^{-06}$ to PfamB1302).	$1e^{-34}$	PfamB252 $2.8e^{-38}$ KOG2504 $2e^{-16}$	Monocarboxylate transporter
PFI1295c Genefinder	9	Membrane transporter, putative	612	11/12	**PFB0465c** (16804992).	$6e^{-30}$	KOG2504 $2e^{-09}$ PfamB252 $8.3e^{-08}$	Monocarboxylate transporter
**Peptide-Acetyl-Coenzyme A Transporter Family** (TC 2.A.1.25; KOG3574)								
PF10_0360 Genefinder	10	Hypothetical protein	~579	9/11	***A.gambiae* hypothetical protein** (31199731; $4e^{-153}$ to KOG3574). Chr10.gen_231 appears to contain ORFs for 3 different proteins, amino acid residues 1644-2222 were used in our analysis.	$1e^{-61}$	KOG3574 $3e^{-69}$	Acetyl-CoA transporter
**Glycoside-Pentoside-Hexuronide:Cation Symporter Family** (TC 2.A.2; COG2211)§								
PFE1455w	5	Sugar transporter, putative	548	12	***C. aurantiacus* hypothetical protein** (22972266; $4e^{-90}$ to COG2211).	$9e^{-20}$	COG2211 $1e^{-30}$	Sugar: cation symporter
**Organo Anion Transporter Family** (TC 2.A.60; Pfam03132, KOG3626)§								
MAL6P1.283	6	Hypothetical protein	853	12	***A.thaliana* hypothetical protein** (4314370; $7e^{-107}$ to KOG1330 and $3e^{-11}$ to KOG3626).	$1.9e^{-08}$	Pfam03132 $7.8e^{-05}$ KOG1330 $1e^{-07}$	Organic anion transporter
**Folate-Biopterin Transporter Family** (TC 2.A.71; Pfam03092)§								
MAL8P1.13	8	Integral membrane protein, conserved	505	12	**PF11_0172** (23508363).	$8e^{-50}$	Pfam03092 $3.9e^{-35}$	Folate/biopterin transporter
PF10_0215 GlimmerM	10	Hypothetical protein	629	12/11	***T. elongatus* hypothetical protein** (22297560; $2.8e^{-164}$ to Pfam03092).	$6e^{-04}$	Pfam03092 0.079 (p)	Metabolite/vitamin transporter
PF11_0172	11	Hypothetical protein, conserved	455	11	***C. parvum* putative biopterin transporter** (32398782; $4.2e^{-56}$ to Pfam03092).	$7e^{-95}$	Pfam03092 $7.8e^{-44}$	Folate/biopterin transporter
**Novel Putative Transporter Family**								
PFA0240w	1	Hypothetical protein	711	12/11	**PFA0245w** (23613408).	$4.9e^{-59}$	No matches	Transporter
PFA0245w	1	Hypothetical protein	577	12	**PF11_0310** (23508501).	$2e^{-28}$	KOG0569 0.008 (p)	Transporter
PFC0530w	3	Hypothetical protein	579	12/11	**PFA0245w** (23613408).	$3.5e^{-07}$	No matches	Transporter
PFI0720w	9	Hypothetical protein	516	11	**PFA0245w** (23613408).	$5e^{-15}$	No matches	Transporter
PF11_0310	11	Hypothetical protein	609	12	**PFA0240w** (23513407).	$1.1e^{-34}$	No matches	Transporter

**Zinc (Zn^{2+})-iron (Fe^{2+}) Permease family** (TC 2.A.5; KOG1558)								
MAL6P1.94	6	Transporter protein, putative	361	8	***D. melanogaster* hypothetical protein** (24586056; $1e^{-58}$ to KOG1558).	$1e^{-10}$	KOG1558 $1e^{-13}$	Zn^{2+} or Fe^{2+} permease
**Resistance-Nodulation-Cell Division Superfamily** (TC 2.A.6; COG1033)								
**Eukaryotic (Putative) Sterol Trransporter Family** (TC 2.A.6.6; KOG1934)								
PFA0375c	1	Patched family protein, putative	1470	12	***C. parvum* putative patched family protein** (46227691; $3e^{-56}$ to KOG1934).	$1e^{-38}$	KOG1934 $2e^{-41}$	Lipid/sterol:H^+ symporter
**Drug/Metabolite Transporter Superfamily** (TC 2.A.7; Pfam00892, KOG3912)								
PF07_0070	7	Transporter/permease protein, putative	322	9	***G. lamblia* hypothetical protein** (29247301; $4e^{-07}$ to KOG3912).	$9e^{-20}$	KOG3912 $3e^{-04}$ Pfam00892 0.008 (p)	Drug/metabolite transporter
PFE0785c	5	Hypothetical protein	456	10	***D. discoideum* putative GDP-fucose: GMP antiporter** (28828290; $5e^{-62}$ to KOG1442).	$2e^{-04}$	No matches	Drug/metabolite transporter
**Triose-phosphate Transporter Family** (TC 2.A.7.9; KOG1441, KOG1443)								
PFE0410w*	5	Triose- or hexose-P_i: P_i antiporter, putative	342	9	***P. sativum* chloroplast triose-P_i: P_i antiporter** (117290; $2e^{-46}$ to KOG1441).	$1e^{-47}$	KOG1441 $4e^{-29}$	Triose- or hexose-P_i:P_i antiporter
PFE1510c*	5	Phosphoenolpyruvate: P_i antiporter, putative	524	7/8	**PFE0410w** (2361306).	$3e^{-44}$	KOG1441 $1e^{-30}$	Phosphoenolpyruvate:P_i antiporter
PFL0890c	12	Hypothetical protein	491	10	***S. bicolor* putative phosphoenolpyruvate: phosphate antiporter** (18481711; $7e^{-40}$ to KOG1441).	$5e^{-13}$	KOG1443 $6e^{-06}$	Triose- or hexose-P_i:P_i antiporter
**GDP-Mannose: GMP Antiporter Family** (TC 2.A.7.13; KOG1442)								
PFB0535w *gi:7494384*	2	Probable multiple-TM membrane protein Hypothetical protein	311	7/8	***R. norvegicus* putative GDP-fucose: GMP antiporter** (34856560; $1e^{-136}$ to KOG1442).	$2e^{-16}$	KOG1442 $9e^{-08}$	GDP-fucose:GMP antiporter
**UDP-N-acetyl glucosamine: UMP Antiporter Family** (TC 2.A.7.10; KOG2234)								
PFE0260w	5	Hypothetical protein	611	9/10	***R. norvegicus* putative NDP-sugar: NMP antiporter** (34859909; $8e^{-104}$ to KOG2234).	$3e^{-10}$	KOG2234 $4e^{-11}$	UDP-N-acetyl glucosamine:UMP antiporter
**UDP-galactose: UMP Antiporter Family** (TC 2.A.7.11; KOG1581)								
PF11_0141	11	UDP-galactose transporter, putative	343	8/9	***R. prolixus* putative UDP-galactose: UMP antiporter** (33518713; $8e^{-48}$ to KOG1581).	$3e^{-33}$	KOG1581 $3e^{-36}$	UDP-galactose:UMP antiporter
**10 TMS Drug/metabolite Exporter Family** (TC 2.A.7.3; COG0697, Pfam00892, KOG3912)								
PF07_0064	7	Hypothetical protein	434	9/10	***B. anthracis* putative transporter** (30260969; $3e^{-08}$ to Pfam00892).	$2e^{-13}$	COG0697 $1e^{-08}$	Drug/metabolite exporter
MAL7P1.27	7	Chloroquine resistance transporter, putative	424	10	***C. parvum* putative transporter** (32399066; Pfam00892 0.049 (p)).	$1e^{-25}$	KOG3912 $4e^{-04}$	Chloroquine resistance transporter (PfCRT; [17, 19]])
**Amino Acid/Auxin Permease Family** (TC 2.A.18; Pfam01490)								
MAL6P1.133	6	Transmembrane amino acid transporter protein, putative	606	9/11	***S. pombe* putative amino acid transporter** (3367790; $1.2e^{-67}$ to Pfam01490).	$6e^{-13}$	Pfam01490 $2e^{-16}$	Amino acid transporter
PFL0420w	12	Hypothetical protein	1564	10/11	***D. discoideum* hypothetical protein** (28828536; $3e^{-04}$ to Pfam01490).	$3e^{-28}$	Pfam01490 $7e^{-11}$	Amino acid transporter
PFL1515c*	12	Hypothetical protein	1936	9/10	**PFL0420w** (23496616).	$2.1e^{-52}$	Pfam01490 $4e^{-04}$	Amino acid transporter
**Ca^{2+}:Cation Antiporter Family** (TC 2.A.19; KOG1397)								
MAL6P1.38	6	Calcium antiporter, putative	441	11/10	***N. crassa* putative Ca^{2+}:H^+ antiporter** (32413763; $3e^{-86}$ to KOG1397).	$2e^{-73}$	KOG1397 $3e^{-65}$	Ca^{2+}:H^+ antiporter
**Inorganic Phosphate Transporter Family** (TC 2.A.20; Pfam01384)								
MAL13P1.206	13	Phosphate transporter, putative	687	10/11	***T. chui* high affinity phosphate transporter** (28569257; $7.2e^{-143}$ to Pfam01384).	$1.7e^{-95}$	Pfam01384 $1.4e^{-114}$	Na^+-dependent P_i transporter†
**Neurotransmitter:Na^+ Symporter Family** (TC 2.A.22; Pfam00209, KOG3660)								
PFB0435c *gi:16804986*	2	Predicted amine transporter Hypothetical protein	1138	14/11	***D. melanogaster* hypothetical protein** (24661428; $1e^{-163}$ to KOG3660).	$9e^{-16}$	KOG3660 $4e^{-25}$ Pfam00209 $2e^{-07}$	Amino acid transporter
PFE0775c	5	Hypothetical protein	960	14/15	**PFB0435c** (7494357).	$3.5e^{-20}$	No matches	Amino acid transporter
PF11_0334 *GlimmerM*	11	Hypothetical protein	1439	15/14	***H. sapien* GABA transporter** (4507039; 0.0 to Pfam00209).	$6e^{-06}$	Pfam00209 $1.3e^{-05}$	Amino acid transporter

**Monovalent Cation:H^{+} Antiporter-1 Family** (TC 2.A.36; Pfam00999)								
PF13_0019	13	Na^{+}:H^{+} exchanger, putative	1920	14	***C. parvum* putative Na^{+}:H^{+} antiporter** (32398656; 1.4e^{-48} to Pfam00999).	2e^{-64}	Pfam00999 3e^{-28}	Na^{+}:H^{+} antiporter
**Formate-Nitrite Transporter Family** (TC 2.A.44; COG2116)								
PFC0725c	3	Transporter, putative	309	6/7	***B. anthracis* putative formate-nitrite transporter** (21401706; 2e^{-50} to COG2116).	5e^{-25}	COG2116 4e^{-35}	Formate-nitrite transporter
**Sulphate Permease Family** (TC 2.A.53; COG0659)								
PF14_0679	14	Sulphate transporter, putative	664	11	***V. cholerae* putative sulphate permease** (9656576; 7e^{-90} to COG0659).	7.2e^{-66}	COG0659 7e^{-37}	Inorganic anion exchanger
**Metal Ion (Mn^{2+}-iron) Transporter Family** (TC 2.A.55; KOG1291)								
PFE1185w	5	Transporter, putative	684	11	***A. thaliana* NRAMP metal ion transporter 4** (15240711; 2e^{-169} to KOG1291).	9e^{-52}	KOG1291 5e^{-59}	Divalent cation (metal) :H^{+} symporter
**Equilibrative Nucleoside Transporter Family** (TC 2.A.57; KOG1479)								
PFA0160c	1	Integral membrane protein	460	11	**MAL8P1.32** (23612771) shares limited similarity in sequence with PFA0160c and is a structural homolog of this protein.	0.38	No matches	Nucleoside transporter
MAL8P1.32	8	Nucleoside transporter, putative	585	10/11	**PF13_0252 PfENT1** (7688921).	4e^{-12}	No matches	Nucleoside transporter
PF13_0252	13	Nucleoside transporter 1	422	9/10	***T. gondii* putative adenosine transporter** (6062551; 2e^{-11} to KOG1479).	3.1e^{-11}	KOG1479 2e^{-05}	Nucleoside transporter 1 (PfENT1; [62, 63])
PF14_0662	14	Hypothetical protein	437	11	The ***D. melanogaster* hypothetical protein** (24580625; 5e^{-78} to KOG1479) shares limited similarity in sequence with PF14_0662 and is a structural homolog of this protein.	0.037	KOG1479 3e^{-11}	Nucleoside transporter
**Multi Antimicrobial Extrusion (MATE) Family** (TC 2.A.66.1; COG0534)								
PFB0580w	2	Hypothetical protein	1224	11/10	***A. fumigatus* putative multidrug:Na^{+} antiporter** (21627817; 1e^{-60} to COG0534).	7e^{-08}	COG0534 6e^{-07}	MATE antiporter
**Voltage-gated Ion Channel Superfamily** (TC 1.A.1; KOG1420, KOG4390)								
PFL1315w	12	Hypothetical protein	1979	8/10	***Nostoc sp* putative ion transporter** (17232125; 3e^{-15} to KOG4390 and 4e^{-08} to KOG1420).	5e^{-18}	KOG1420 4e^{-09}	K^{+} channel
PF14_0342*	14	Hypothetical protein	1898	5/12	**PFL1315w** (AAN36349).	2.2e^{-58}	No matches	Ion channel
PF14_0622	14	Hypothetical protein	1495	9/11	***Nostoc sp* putative ion transporter** (17232125).	3e^{-08}	KOG1420 3e^{-06}	K^{+} channel
**ATP-binding Cassette (ABC) Superfamily** (TC 3.A.1; KOG0059, COG1129)								
PFC0875w	3	Hypothetical protein	3032	12/14	***D. discoideum* putative ABC transporter** (19110836; 1e^{-65} to KOG0059).	4.5e^{-16}	COG1129 6e^{-14}	ABC transporter
**MHC Peptide Transporter (TAP) Family** (TC 3.A.1.209 (DPL); KOG0058)								
PFC0125w*,‡	3	ABC transporter, putative	1365	6	***C. tepidum* putative ABC transporter** (21674321; 1e^{-105} to KOG0058).	3e^{-22}	KOG0058 4e^{-23}	ABC transporter (TAP family)
PF11_0466*	11	Transport protein, putative	872	8	***D. rerio* putative TAP protein** (26788076; 7e^{-142} to KOG0058).	9e^{-32}	KOG0058 7e^{-37}	ABC transporter (TAP family)
PFL0495c‡	12	ABC transporter, putative	855	5/6	***H. sapien* putative MDR/TAP protein** (9961244; 0.0 to KOG0058).	1e^{-45}	KOG0058 8e^{-67}	ABC transporter (TAP family)
**Multidrug Resistance Exporter (MDR) Family** (TC 3.A.1.201 (DPL); KOG0055)								
PFE1150w	5	Multidrug resistance protein	1419	11	***R. norvegicus* putative multidrug/pheromone exporter Mdr1a** (25453402; 0.0 to KOG0055).	e^{-154}	KOG0055 0.0	Multidrug resistance protein (MDR family) (PfMDR1; [124, 125])
PF13_0218‡	13	ABC transporter, putative	925	4/6	***S. histriomuscorum* putative ABC transporter** (13346110; 2e^{-140} to KOG0055).	3e^{-21}	KOG0055 2e^{-22}	ABC transporter (MDR family)
**Heavy Metal Transporter (HMT) Family** (TC 3.A.1.210 (DPL); KOG0056, KOG0057)								
PF13_0271*,‡	13	ABC transporter, putative	1049	5/6	***A. thaliana* putative ABC transporter** (15237155; 0.0 to KOG0057).	7e^{-59}	KOG0057 2e^{-41}	ABC transporter (HMT family)
PF14_0455	14	Multidrug resistance protein 2	1024	10/11	***C. elegans* ABC transporter required for heavy metal detoxification** (25150042; 0.0 to KOG0056).	e^{-112}	KOG0056 4e^{-109}	Multidrug resistance protein 2 (HMT family) (PfMDR2; [126])
**Conjugate Transporter (CT) Family** (TC 3.A.1.208 (OAD); KOG0054)								
PFA0590w	1	ABC transporter, putative	1822	11/13	**PFL1410c** (23508976).	e^{-160}	KOG0054 4e^{-45}	ABC transporter (CT family) (PfMRP; [127])
PFL1410c	12	Hypothetical protein	2108	11/17	**PFA0590w** (23613477).	e^{-164}	KOG0054 2e^{-22}	ABC transporter (CT family)
**Eye Pigment Precursor Transporter (EPP) Family** (TC 3.A.1.204 (EPD); KOG0061)								
PF14_0244‡	14	ABC transporter, putative	660	6	***A. thaliana* putative ABC transporter** (18415230; 3e^{-131} to KOG0061).	4e^{-71}	KOG0061 2e^{-59}	ABC transporter (EPP family)
**P-type ATPase (P-ATPase) Superfamily** (TC 3.A.3; KOG0202, KOG0204, KOG0206-10)								
PFA0310c	1	Calcium-transporting ATPase	1228	8/11	***L. esculentum* Ca^{2+}-transporting P-type ATPase LCA1** (170378; 0.0 to KOG0202).	e^{-154}	KOG0202 3e^{-169}	SERCA-type Ca^{2+}-transporting P-ATPase (PfATPase6; [128-130])

PFL0590c	12	P-type ATPase, putative	1208	8	***M. barkeri* putative cation-transporting P-type ATPase** (23051256; 0.0 to KOG0202).	e^{-125}	KOG0202 $4e^{-152}$	Non-SERCA-type Ca^{2+}-transporting P-ATPase (PfATPase4; [130, 131])
MAL13P1.246	13	Hypothetical protein	1828	9/11	***T. gondi* Ca^{2+}-transporting P-type ATPase** (8100498; $5e^{-140}$ to KOG0204).	$1e^{-11}$	KOG0204 $6e^{-06}$	Cation-transporting P-ATPase
PFI0240c	9	E1-E2_ATPase/ hydrolase putative	2563	6/10	***C. parvum* putative Cu^{2+}-transporting P-type ATPase** (10122140; $2e^{-36}$ to KOG0207).	$1e^{-29}$	KOG0207 $2e^{-28}$	Cu^{2+}-transporting P-ATPase (PfCuP-ATPase; [132])
PFE0195w	5	P-type ATPase, putative	2393	12/14	***C. parum* putative Ca^{2+}-transporting P-type ATPase** (7021535; $5e^{-36}$ to KOG0208).	$2e^{-40}$	KOG0208 $2e^{-41}$	Cation-transporting P-ATPase (PfATPase3; [133])
PFE0805w*	5	Cation-transporting ATPase 1	2400	10/12	***M. musculus* putative cation-transporting P-type ATPase** (31982093; 0.0 to KOG0208).	$4e^{-40}$	KOG0208 $3e^{-44}$	Cation-transporting P-ATPase (PfATPase1; [134])
PF07_0115*	7	Integral membrane protein, putative	1918	12/13	***A. gambiae* hypothetical protein** (31223925; 0.0 to KOG0209).	$8e^{-56}$	KOG0209 $4e^{-62}$	Cation-transporting P-ATPase
PF11_0395	11	Guanylyl cyclase	4226	19/24	***A. thaliana* putative phospholipid-transporting P-type ATPase II** (15222212; 0.0 to KOG0206).	$3e^{-60}$	KOG0206 $2e^{-80}$	Protein with aminophospholipid-transporting P-ATPase and guanylyl cyclase domains
PF13_0320	13	Guanylyl cyclase	3127	21/22	**PF11_0395** (23508585).	$3e^{-53}$	KOG0206 $3e^{-23}$	Protein with aminophospholipid-transporting P-ATPase and guanylyl cyclase domains
PFC0840w	3	P-type ATPase, putative	1864	10/11	***A. gambiae* hypothetical protein** (31206633; $4e^{-134}$ to KOG0206).	$2e^{-61}$	KOG0206 $4e^{-62}$	Aminophospholipid-transporting P-ATPase (PfATPase7; [135])
PFL0950c	12	P-type ATPase2	1555	10/11	***D. discoideum* hypothetical protein** (28850447; 0.0 to KOG0206).	e^{-100}	KOG0206 $8e^{-134}$	Aminophospholipid-transporting P-ATPase (PfATPase2; [136])
PFL1125w	12	Phospholipid-transporting ATPase, putative	1618	10	***M. musculus* putative phospholipid-transporting P-type ATPase IIB** (29839753; 0.0 to KOG0202).	e^{-114}	KOG0210 $3e^{-104}$	Aminophospholipid-transporting P-ATPase
PF14_0654	14	Hypothetical protein	1837	6/8	**PFL1125w** (23508919).	$4.8e^{-33}$	KOG0210 $4e^{-08}$	Aminophospholipid-transporting P-ATPase
**H^+-TRANSLOCATING PYROPHOSPHATASE FAMILY** (TC 3.A.10; PFAM03030)								
PFL1700c	12	V-type H^+-translocating inorganic pyrophosphatase	1044	16	***A. thaliana* vacuolar-type H^+-translocating inorganic pyrophosphatase** (6901676; 0.0 to Pfam03030).	$1.1e^{-226}$	Pfam03030 $9.2e^{-247}$	V-type K^+-independent H^+-translocating inorganic pyrophosphatase (PfVP2; [23])
PF14_0541	14	V-type H^+-translocating pyrophosphatase, putative	717	16/15	***T. gondii* H^+-translocating inorganic pyrophosphatase 1** (6901676; 0.0 to Pfam03030).	0.0	Pfam03030 0.0	V-type K^+-dependent H^+-translocating inorganic pyrophosphatase (PfVP1; [23])

[a] Italics indicate where an alternate gene model was used in place of the Gardner et al. (2002) [3] version. Gene products were assigned to a known transport family according to the official transporter classification system [10]. The Transport Commission (TC) number and examples of conserved domains of the family are shown in parentheses. Abbreviations are as follows: COG, clusters of orthologous groups of proteins from prokaryotes and unicellular eukaryotes [137]; KOG, clusters of orthologous groups of proteins from eukaryotes [138]; Pfam, protein families and domains [139]; DPL, drugs, peptides, lipids; OAD, organic anion conjugates, anions, drugs; EPD, eye pigment precursors and drugs.

[b] Chromosome on which the gene is located.

[c] For some chromosome 2 proteins the annotation given by Gardner et al. (2002) [3] differed from that of Gardner et al. (1998) [140], in which case the 2002 annotation is listed first and the 1998 second.

[d] Amino acid residues.

[e] Number of transmembrane domains predicted to be present in the protein. Each protein was analysed by the TMHMM2 and TMPRED programs and where the outputs from the two programs were dissimilar, the predicted number of transmembrane domains were tabulated as TMHMM2/TMpred.

[f] The (non-*P. yoelii)* protein that was retrieved with the highest sequence similarity to the *P. falciparum* protein in a BLASTP search of the NCBI non-redundant protein database. The NCBI (gi) accession number and the best match to a conserved domain for the retrieved protein are given in parentheses. Abbreviations are as follows: MFS, major facilitator superfamily; NRAMP, 'natural resistance-associated' macrophage protein.

[g] The ‘E’ value is the expectation that the sequence similarity shared by the *P. falciparum* protein and the protein identified in the Table as its ‘Closest BLAST homologue’ arose by chance. An ‘E’ value $< 10^{-04}$ is considered to indicate a significant sequence similarity between the two proteins, consistent with their having a related biological function.

[h] E value for the best conserved domain match to the *P. falciparum* protein. (p) indicates that the match is only putative as the E value is $= 10^{-03}$.

[i] Functions have been assigned (tentatively) on the basis of the specificity and transport mechanism (proven or postulated) of related proteins as well as similarities to conserved domains.

* These proteins are predicted to contain an apicoplast targeting signal ([14]; GO cellular component ‘apicoplast (GO:0020011)’; PlasmoDB). None of the proteins presented in Tables 1-3 are predicted to be targeted to the mitochondria.

§ These families are related to the MFS.

† R. Martin, K. Saliba, A. Bröer, C. McCarthy, M. Downie, R. Henry, R. Allen, S. Bröer and K. Kirk, manuscript in preparation.

‡ These putative ABC transport proteins were not retrieved by the hydropathy-based approach, but have been included to complete the overview of ABC transport proteins in *P. falciparum*.
