# Supplementary material for: The 'permeome' of the malaria parasite: an overview of the membrane transport proteins of Plasmodium falciparum
Source: Genome Biol. 2005 Mar 2;6(3):R26. doi: 10.1186/gb-2005-6-3-r26 (PMC1088945; doi:10.1186/gb-2005-6-3-r26)
Supplement: Additional File 2 — Proteins that are related to 'hypothetical proteins' from other organisms and have predicted secondary structures that resemble those of characterized transport proteins, but which do not share sequence similarities with known or putative transport proteins and/or conserved domains of transport protein families [file gb-2005-6-3-r26-S2.pdf]

Table 2: *P. falciparum* putative transport proteins: proteins that are related to 'hypothetical proteins' from other organisms and have predicted secondary structures that resemble those of characterised transport proteins, but which do not share sequence similarities with known or putative transport proteins and/or conserved domains of transport protein families.

Gene Product[a]	Ch[b]	Annotation[c]	Size[d]	TMD[e]	Closest BLASTP Homologue[f]	E[G]	Conserved Domain Match[h]
PFE1130w	5	Hypothetical protein	483	9/10	***A. thaliana* hypothetical protein** (15231596; $5e^{-77}$ to KOG2922).	$4e^{-13}$	KOG2922 $5e^{-17}$
MAL6P1.277	6	Hypothetical protein	1096	11/10	***C. elegans* hypothetical protein** (17554238; 0.0 to KOG2365).	$7e^{-16}$	KOG2365 $8e^{-12}$
MAL6P1.292	6	Integral membrane protein	1347	7/8	***M. musculus* integral membrane protein** (31981342; $2.8e^{-141}$ to Pfam06762).	$3e^{-29}$	Pfam06762 $2e^{-21}$
MAL8P1.30	8	Hypothetical protein	643	10/11	***M. musculus* integral membrane protein** (31981342).	$7e^{-10}$	No matches
MAL8P1.135	8	Hypothetical protein	965	9/11	***H. sapien* hypothetical protein** (37538400; 0.0 to KOG4587).	$2e^{-19}$	KOG4587 $1e^{-06}$
PFL2410w*	12	Hypothetical protein	1039	9/10	***R. norvegicus* integral membrane protein** (34867474; $3e^{-62}$ to KOG1134).	$1e^{-19}$	KOG1134 $6e^{-15}$
PF13_0172*	13	Hypothetical protein	522	11/10	***A. thaliana* hypothetical protein** (15237867; $.2e^{--222}$ to Pfam 05684).	$2e^{-05}$	Pfam05684 $1e^{-09}$
**Apicomplexa-Specific Family of Putative Transport Proteins**							
PFB0770c	2	Hypothetical protein	1122	15/15	***C. parvum* hypothetical protein** (46227024; no matches).	$2e^{-26}$	No matches
PFI1205c	9	Hypothetical protein	1249	13/12	***C. parvum* hypothetical protein** (46227024; no matches).	$2e^{-07}$	No matches

[a-h,*] as described in Additional data file 1.
