# Supplementary material for: The 'permeome' of the malaria parasite: an overview of the membrane transport proteins of Plasmodium falciparum
Source: Genome Biol. 2005 Mar 2;6(3):R26. doi: 10.1186/gb-2005-6-3-r26 (PMC1088945; doi:10.1186/gb-2005-6-3-r26)
Supplement: Additional File 3 — Plasmodium-specific proteins that have the (predicted) structural characteristics of a transporter, but which, apart from strong sequence similarities to hypothetical proteins from other Plasmodium species, do not display any similarities to proteins or conserved domains in the current databases [file gb-2005-6-3-r26-S3.pdf]

Table 3: *P. falciparum* putative transport proteins: *Plasmodia*-specific proteins that have the (predicted) structural characteristics of a transporter, but which, apart from strong sequence similarities to hypothetical proteins from other *Plasmodia* species, do not display any similarities to proteins or conserved domains in the current databases. These proteins do have, to varying degrees, predicted secondary structures that are similar to those of characterised transport proteins, which suggests that these novel proteins might function as transporters.

GENE PRODUCT[a]	CH[b]	ANNOTATION[c]	SIZE[d]	TMD[e]	DESCRIPTION
PFC0240c	3	Hypothetical protein	955	11	The TMDs of PFC0240c are arranged as 2 clusters, one of 5 and the other of 6, which are separated by a hydrophilic loop. The N-terminal hydrophilic tail is long (~400 residues in length).
PFC0685w	3	Hypothetical protein	913	12/14	The TMDs of PFC0685w are arranged, from N- to C-terminus, as clusters of 3, 4, 1, 4 and 2. The longest hydrophilic loop is between TMDs 8 and 9 (~200 residues in length), the other inter-cluster loops are 60 - 80 residues in length.
PFE1525w*	5	Hypothetical protein	970	10	The leading TMD of the PFE1525w protein is separated from the remaining 9 TMDs by a hydrophilic loop of ~175 residues and the C-terminal tail is long (~300 residues in length).
MAL6P1.144	6	Hypothetical protein	360	8	The TMDs of MAL6P1.144 are in clusters of 2. Unlike most other proteins analysed in this study, MAL6P1.144 does not have an orthologue in *P. yoelii*.
PF11_0363	11	Hypothetical protein	409	8	The TMDs of PF11_0363 are arranged, from N- to C-terminus, as clusters of 1, 2, 2, 2 and 1.

[a-e,*] as described in Additional data file 1.
