# Supplementary material for: The 'permeome' of the malaria parasite: an overview of the membrane transport proteins of Plasmodium falciparum
Source: Genome Biol. 2005 Mar 2;6(3):R26. doi: 10.1186/gb-2005-6-3-r26 (PMC1088945; doi:10.1186/gb-2005-6-3-r26)
Supplement: Additional File 4 — The region over TMDs 1-5 of the alignment is shown. Sequences are separated into clusters of well-related sequences and are grouped as subfamilies of the MFS. P. falciparum sequences are boxed and the protein designators highlighted. For proteins of other organisms, the NCBI accession (gi) number and the known or putative (p) substrate specificity of the transporter are given. In some proteins, one or more of the extramembrane loop regions have been truncated and this is indicated by a solid black line. Residues are shaded as follows: positively charged, blue; negatively charged, red; hydoxyl, orange; amido, grey; proline, green; cysteine, purple; histidine, mid blue; glycine, light blue; tryptophan and tyrosine, olive green; remaining nonpolar, yellow [file gb-2005-6-3-r26-S4.pdf]

MAJOR FACILITATOR SUPERFAMILY

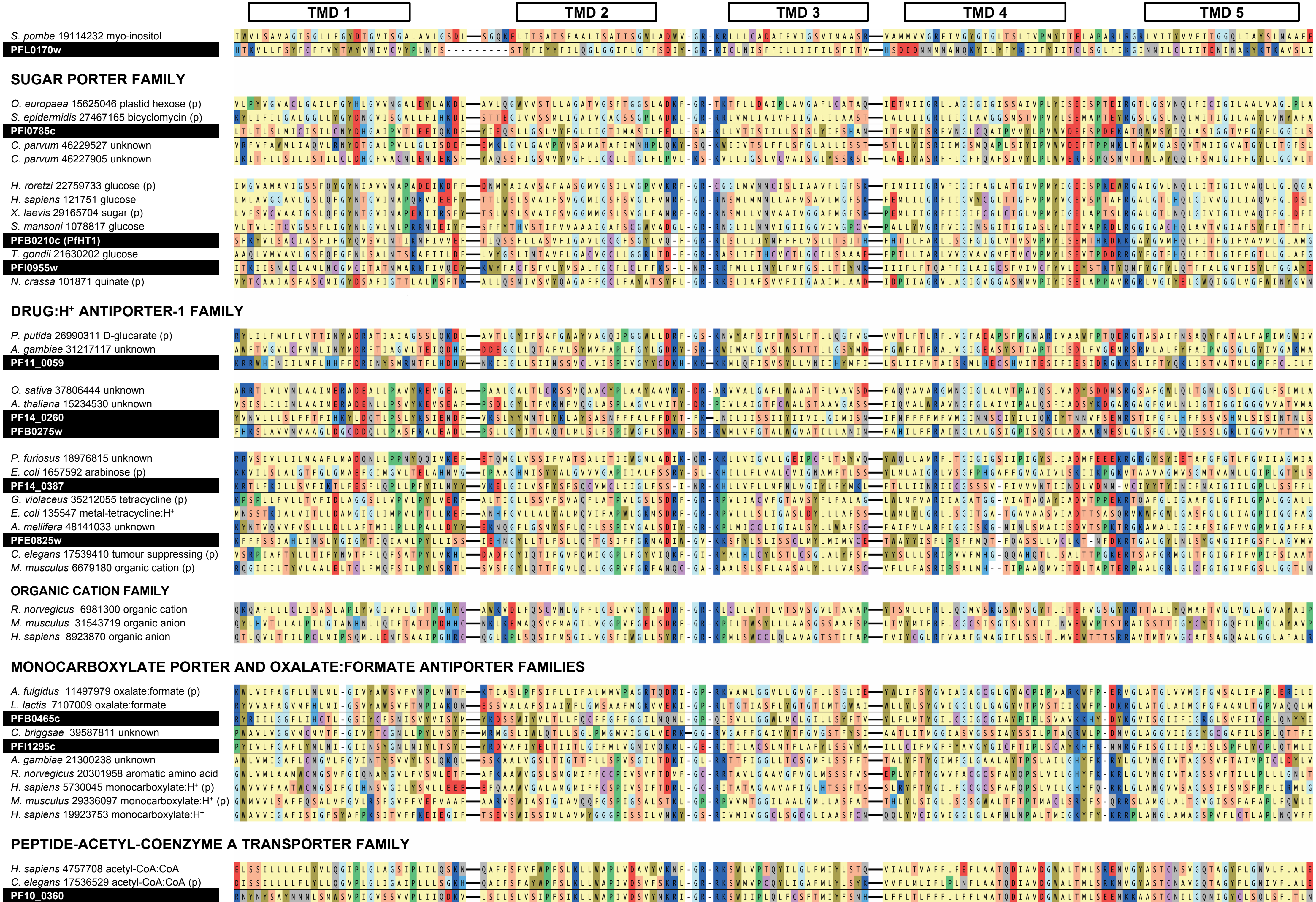

Additional data file 4. The alignment of the *P. falciparum* putative MFS proteins with a representative selection of MFS proteins from other organisms. The region over TMDs 1-5 of the alignment is shown. Sequences are separated into clusters of well-related sequences and are grouped as subfamilies of the MFS. *P . falciparum* sequences are boxed and the protein designators highlighted. For proteins of other organisms, the NCBI accession (gi) number and the known or putative (p) substrate specificity of the transporter are given. In some proteins, one or more of the extramembrane loop regions have been truncated and this is indicated by a solid black line. Residues are shaded as follows: positively charged, blue; negatively charged, red; hydoxyl, orange; amido, grey; proline, green; cysteine, purple; histidine, mid blue; glycine, light blue; tryptophan and tyrosine, olive green; remaining nonpolar, yellow.
