# Supplementary material for: The 'permeome' of the malaria parasite: an overview of the membrane transport proteins of Plasmodium falciparum
Source: Genome Biol. 2005 Mar 2;6(3):R26. doi: 10.1186/gb-2005-6-3-r26 (PMC1088945; doi:10.1186/gb-2005-6-3-r26)
Supplement: Additional File 5 — Both transporter families are distantly related to the MFS. The region over TMDs 2-5 and TMD 8 of the organo anion transporter family alignment is shown. The region over TMDs 3-5 and TMD 10 of the folate-biopterin transporter family alignment is shown. Legend as described for Additional data file 4 [file gb-2005-6-3-r26-S5.pdf]

MFS-RELATED FAMILIES

A. ORGANO ANION TRANSPORTER FAMILY

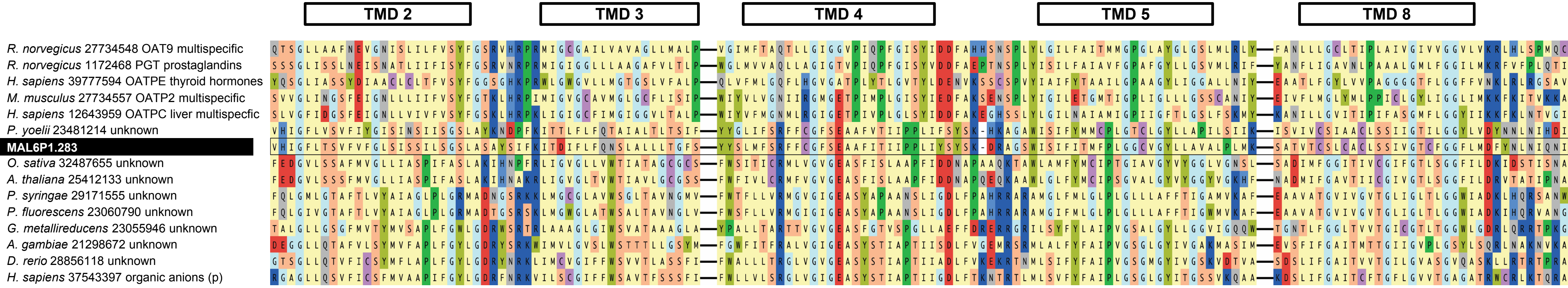

B. FOLATE-BIOPTERIN FAMILY

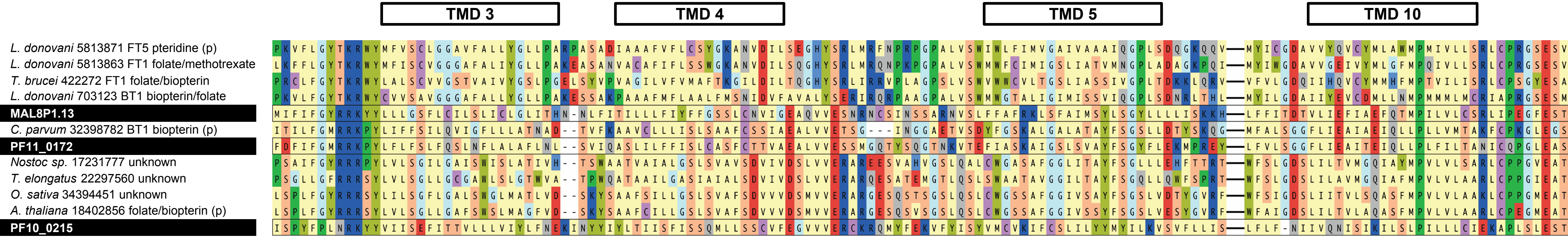

Additional data file 5. The alignment of the *P. falciparum* putative (A) organo anion transporter and (B) folate-biopterin transporters with a representative selection of known and putative organo anion or folate-biopterin transporters, respectively, from other organisms. Both transporter families are distantly related to the MFS. The region over TMDs 2-5 and TMD 8 of the organo anion transporter family alignment is shown. The region over TMDs 3-5 and TMD 10 of the folate-biopterin transporter family alignment is shown. Legend as described for Additional data file 4.
