# Supplementary material for: The 'permeome' of the malaria parasite: an overview of the membrane transport proteins of Plasmodium falciparum
Source: Genome Biol. 2005 Mar 2;6(3):R26. doi: 10.1186/gb-2005-6-3-r26 (PMC1088945; doi:10.1186/gb-2005-6-3-r26)
Supplement: Additional File 6 — (A) Hydropathy plots of two representatives of the novel putative transporter family: the PFI0720w and PFC0530w proteins. The two profiles are very similar and in each, there are 12 clear peaks in hydrophobicity, corresponding to 12 TMDs. A topology of 12 TMDs separated, by an extended extramembrane loop, into two sets of 6 closely spaced TMDs is characteristic of transporters of the MFS. (B) The alignment of the five P. falciparum novel putative transporters. The region over TMDs 2-3 and TMDs 8-9 of the alignment is shown. Members of the MFS family typically possess a conserved amino acid motif between TMDs 2 and 3 and a related but less conserved motif in the corresponding loop in the second half of the protein (between TMDs 8 and 9). The putative novel transport proteins also appear to contain these MFS-specific motifs between TMDs 2 and 3 and, to a lesser extent, between TMDs 8 and 9. For comparison, MFS-specific motifs from a range of known and putative MFS proteins are presented. Legend as described for Additional data file 4 [file gb-2005-6-3-r26-S6.pdf]

FAMILY OF NOVEL PUTATIVE TRANSPORTERS

A. HYDROPATHY PROFILES

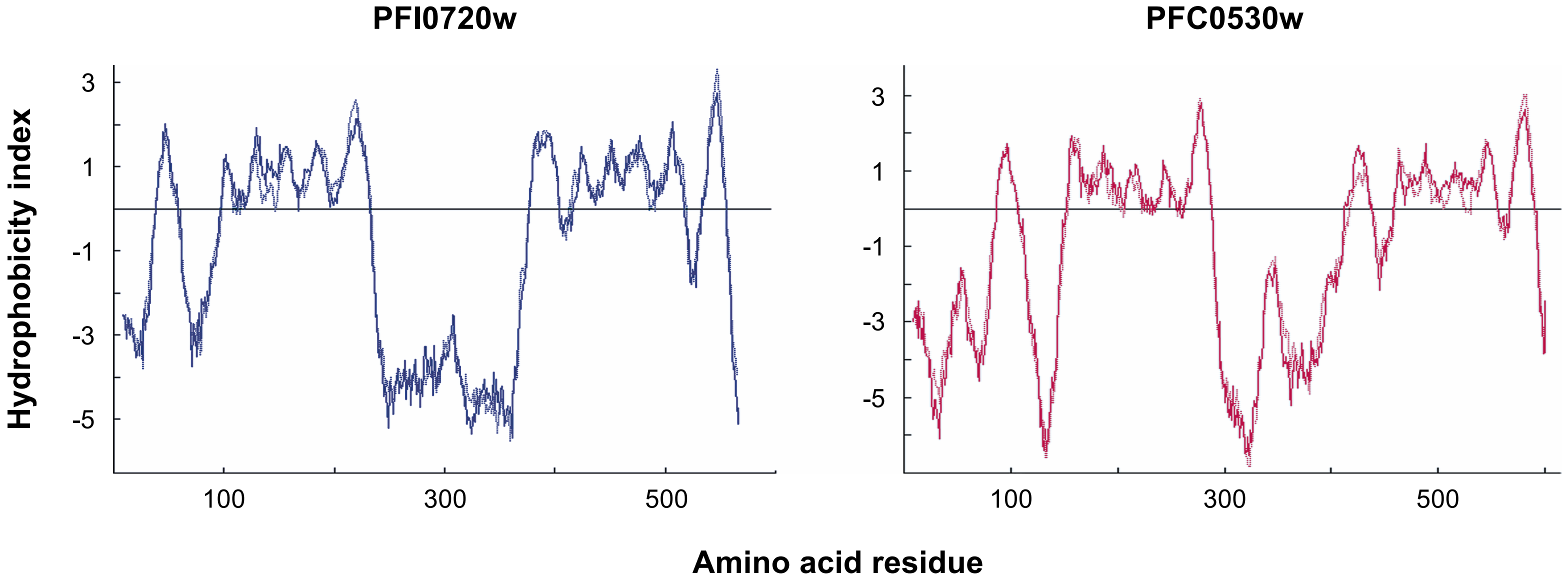

B. SEQUENCE ALIGNMENT OF THE *PLASMODIUM* NOVEL PUTATIVE TRANSPORT PROTEINS

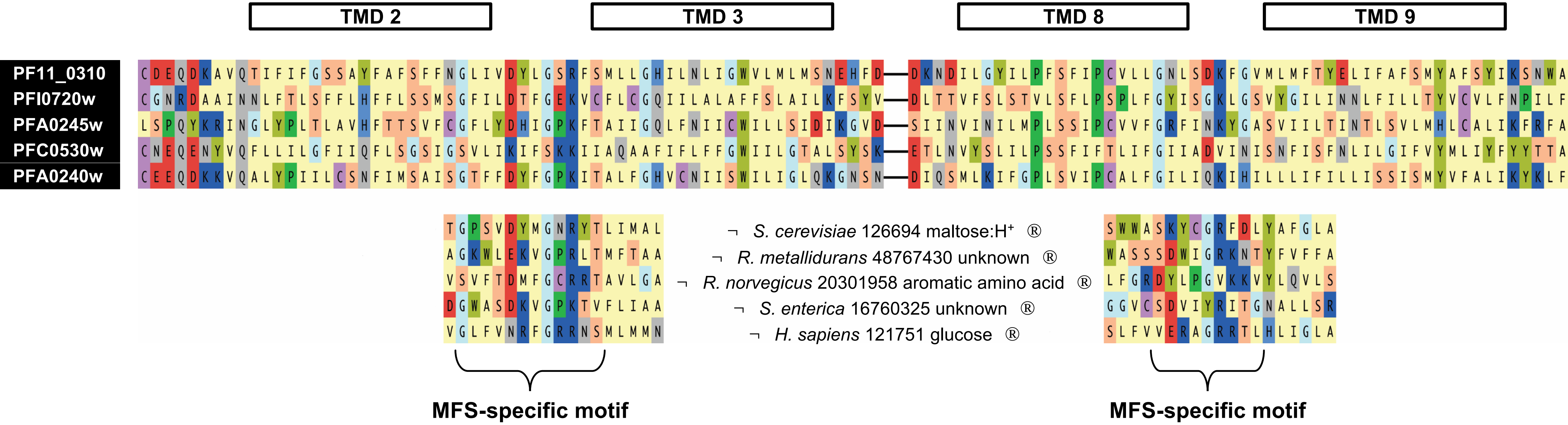

Additional data file 6. The *P. falciparum* family of novel putative transporters. (A) Hydropathy plots of two representatives of the novel putative transporter family: the PFI0720w and PFC0530w proteins. The two profiles are very similar and in each, there are 12 clear peaks in hydrophobicity, corresponding to 12 TMDs. A topology of 12 TMDs separated, by an extended extramembrane loop, into two sets of 6 closely spaced TMDs is characteristic of transporters of the MFS. **(B) The alignment of the five *P. falciparum* novel putative transporters.** The region over TMDs 2-3 and TMDs 8-9 of the alignment is shown. Members of the MFS family typically possess a conserved amino acid motif between TMDs 2 and 3 and a related but less conserved motif in the corresponding loop in the second half of the protein (between TMDs 8 and 9). The putative novel transport proteins also appear to contain these MFS-specific motifs between TMDs 2 and 3 and, to a lesser extent, between TMDs 8 and 9. For comparison, MFS-specific motifs from a range of known and putative MFS proteins are presented. Legend as described for Additional data file 4.
