# Supplementary material for: The 'permeome' of the malaria parasite: an overview of the membrane transport proteins of Plasmodium falciparum
Source: Genome Biol. 2005 Mar 2;6(3):R26. doi: 10.1186/gb-2005-6-3-r26 (PMC1088945; doi:10.1186/gb-2005-6-3-r26)
Supplement: Additional File 7 — The region over TMDs 1-5, TMD 7 and TMD 10 of the alignment is shown. The sequences are separated into two clusters, one containing plant and insect proteins and the other protozoan, yeast and mammalian proteins. The PFL0420w and PFL1515c proteins appear to be more closely related to the former group of transporters, whereas the MAL6P1.133 is more similar to the latter. Legend as described for Additional data file 4 [file gb-2005-6-3-r26-S7.pdf]

AMINO ACID/AUXIN PERMEASE FAMILY

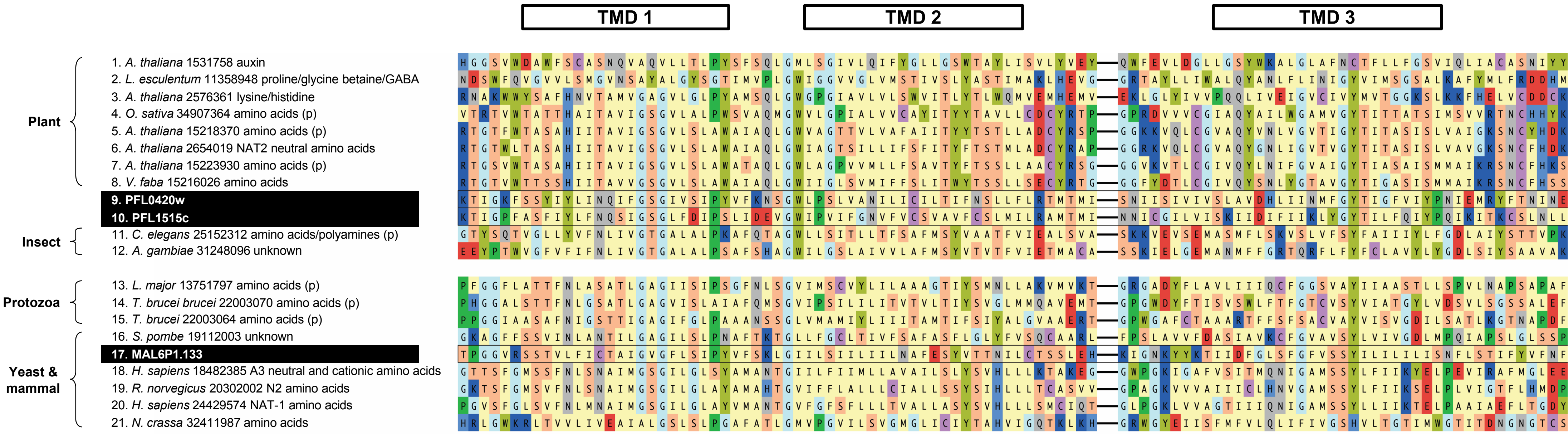

AAAP FAMILY ALIGNMENT CONTINUED …

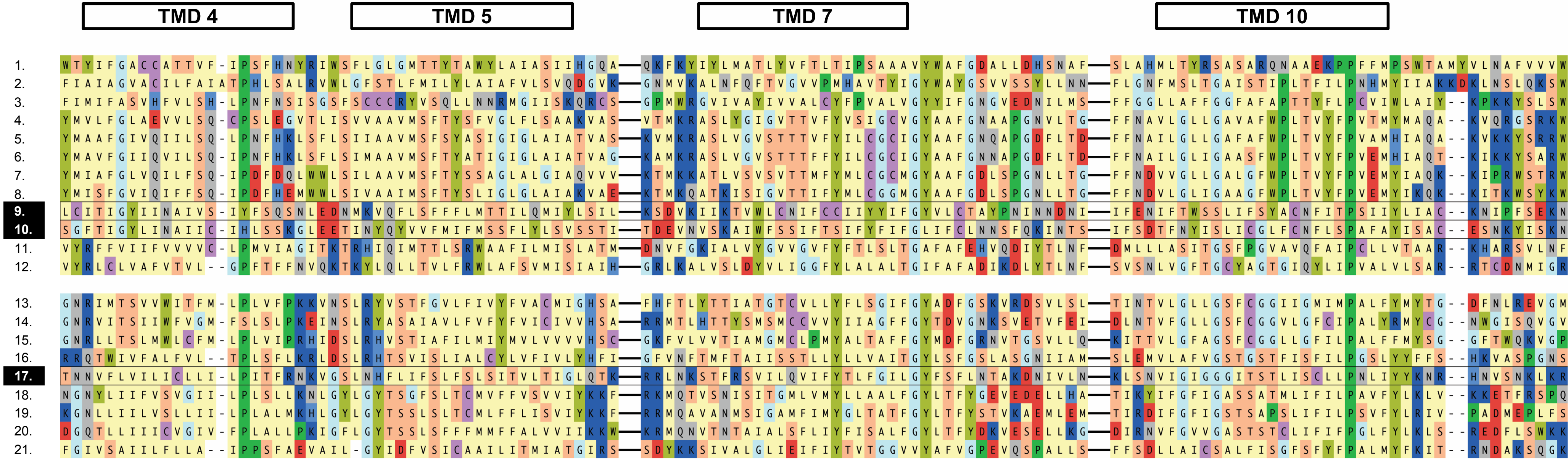

Additional data file 7. The alignment of the *P. falciparum* putative amino acid/auxin transporters with a representative selection of amino acid/auxin transporters (known and putative) from other organisms. The region over TMDs 1-5, TMD 7 and TMD 10 of the alignment is shown. The sequences are separated into two clusters, one containing plant and insect proteins and the other protozoan, yeast and mammalian proteins. The PFL0420w and PFL1515c proteins appear to be more closely related to the former group of transporters, whereas the MAL6P1.133 is more similar to the latter. Legend as described for Additional data file 4.
