# Supplementary material for: The 'permeome' of the malaria parasite: an overview of the membrane transport proteins of Plasmodium falciparum
Source: Genome Biol. 2005 Mar 2;6(3):R26. doi: 10.1186/gb-2005-6-3-r26 (PMC1088945; doi:10.1186/gb-2005-6-3-r26)
Supplement: Additional File 8 — The region over TMDs 1-3 and TMDs 6-10 of the alignment is shown. The sequences are separated into two clusters, one containing proteins known to transport neurotransmitters, creatine or taurine, and the other proteins known or hypothesized to transport amino acids. The P. falciparum proteins have diverged considerably from the other family members, but are perhaps most similar in sequence to the amino acid transporters. Legend as described for Additional data file 4 [file gb-2005-6-3-r26-S8.pdf]

NEUROTRANSMITTER:Na^+ SYMPORTER FAMILY

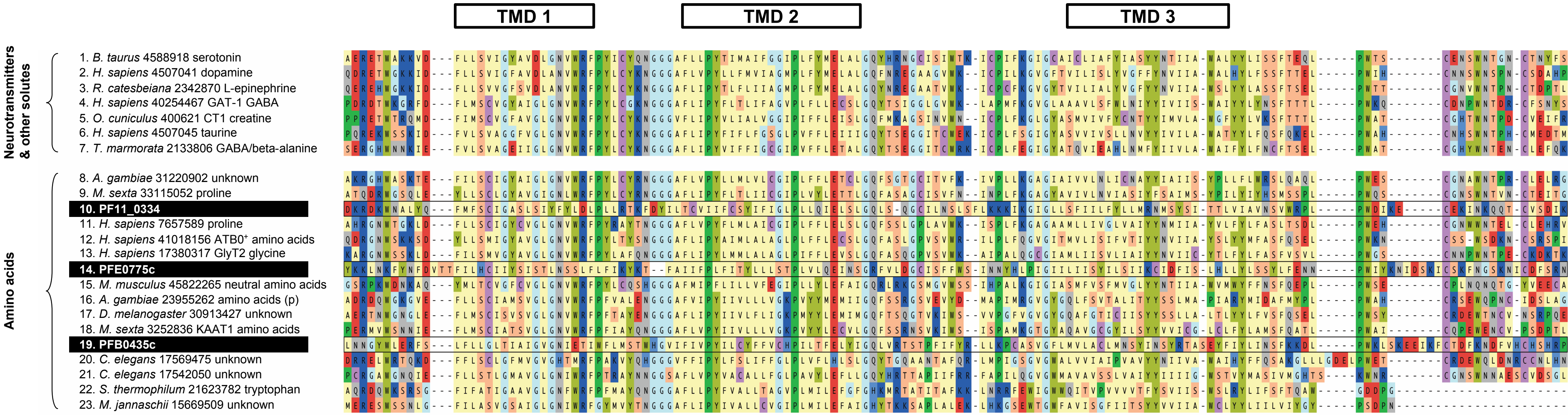

NSS FAMILY ALIGNMENT CONTINUED …

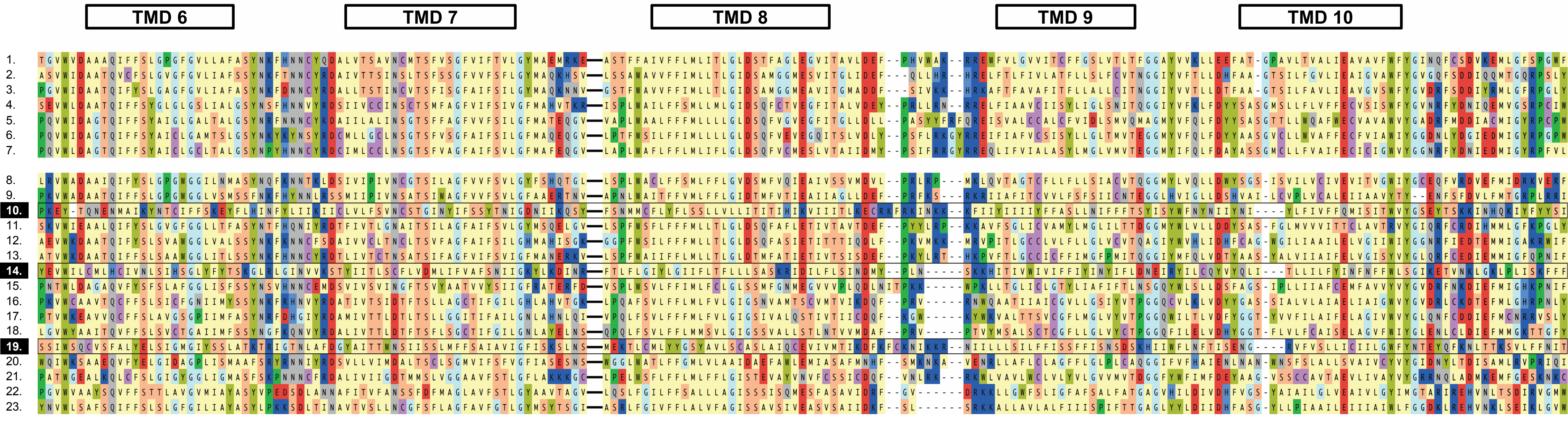

Additional data file 8. The alignment of the *P. falciparum* putative neurotransmitter:Na^+ symporters with a representative selection of neurotransmitter:Na^+ symporters (known and putative) from other organisms. The region over TMDs 1-3 and TMDs 6-10 of the alignment is shown. The sequences are separated into two clusters, one containing proteins known to transport neurotransmitters, creatine or taurine, and the other proteins known or hypothesized to transport amino acids. The *P. falciparum* proteins have diverged considerably from the other family members, but are perhaps most similar in sequence to the amino acid transporters. Legend as described for Additional data file 4.
