# Supplementary material for: The 'permeome' of the malaria parasite: an overview of the membrane transport proteins of Plasmodium falciparum
Source: Genome Biol. 2005 Mar 2;6(3):R26. doi: 10.1186/gb-2005-6-3-r26 (PMC1088945; doi:10.1186/gb-2005-6-3-r26)
Supplement: Additional File 9 — The region over TMDs 2-6 of the alignment is shown. The locations of the 'voltage sensor' and 'selectivity sequence' are indicated. Legend as described for Additional data file 4 [file gb-2005-6-3-r26-S9.pdf]

VOLTAGE-GATED ION CHANNEL SUPERFAMILY

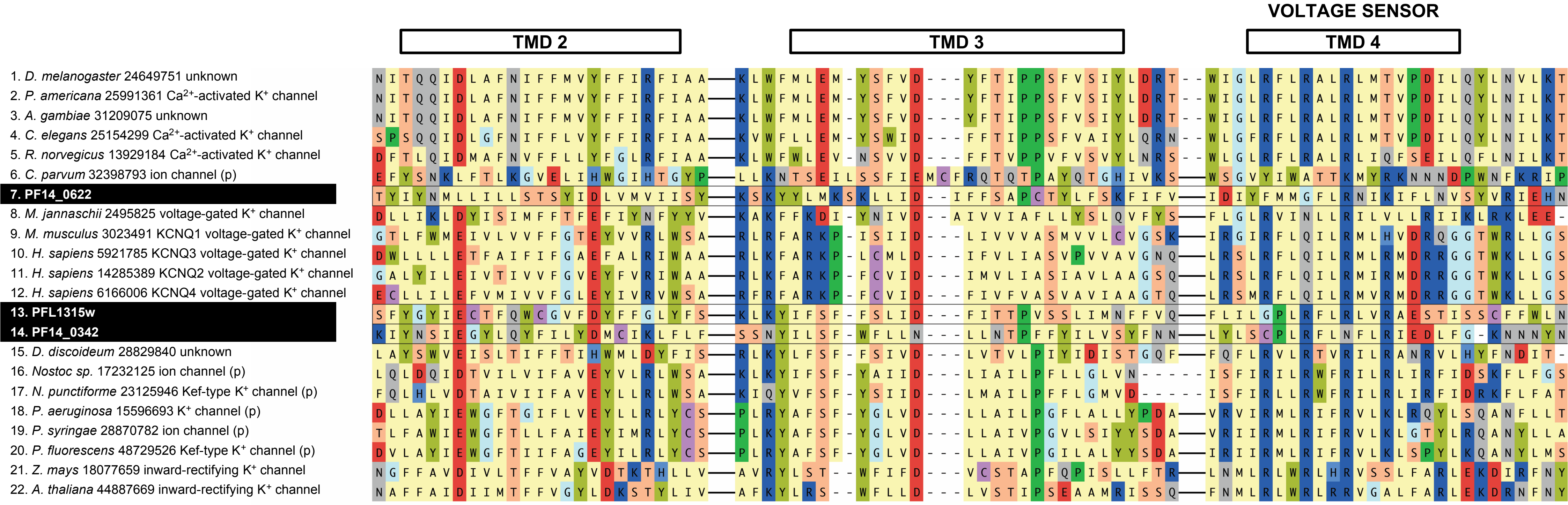

VIC FAMILY ALIGNMENT CONTINUED ...

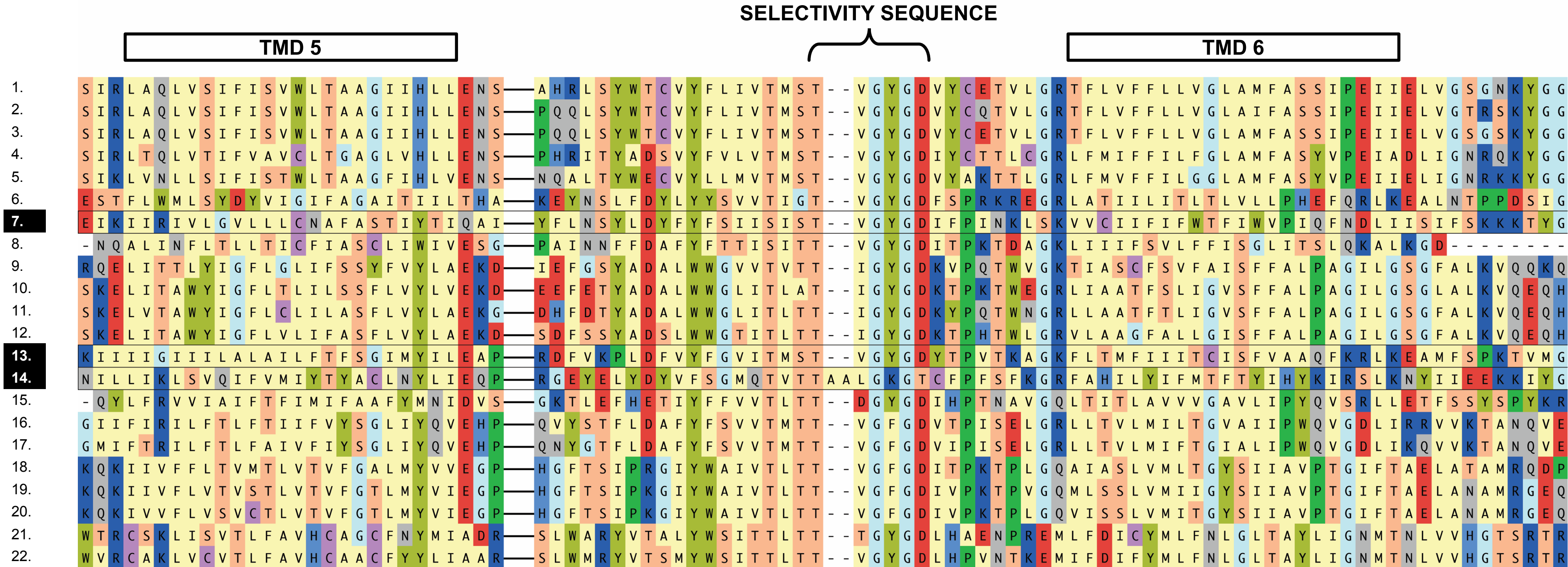

Additional data file 9. The alignment of the putative *P. falciparum* K^+ channel proteins (PFL1315w and PF14_0622) and novel ion channel protein (PF14_0342) with a representative selection of K^+ channel proteins (known and putative) of the voltage-gated ion channel superfamily from other organisms. The region over TMDs 2-6 of the alignment is shown. The locations of the ‘voltage sensor’ and ‘selectivity sequence’ are indicated. Legend as described for Additional data file 4.
