# Supplementary material for: The 'permeome' of the malaria parasite: an overview of the membrane transport proteins of Plasmodium falciparum
Source: Genome Biol. 2005 Mar 2;6(3):R26. doi: 10.1186/gb-2005-6-3-r26 (PMC1088945; doi:10.1186/gb-2005-6-3-r26)
Supplement: Additional File 10 — Additional methods [file gb-2005-6-3-r26-S10.pdf]

ADDITIONAL METHODS

The *P. falciparum* genome was scanned using the following parameters:

Minimum Peptide Length = 250 amino acids
Maximum Peptide Length = 5000 amino acids
Smoothing Window Length = 19 amino acids
Peak Threshold = 1.4 hydropathy units (Kyte-Doolittle)
Valley Threshold = 0.2
Maximum Location for First Peak = within first 1000 amino acids
Minimum Peak Width = 12 amino acids
Maximum Peak Width = 60 amino acids
Allow Merged Peaks = True
Minimum Number of Transmembrane Domains = 7
Maximum Number of Transmembrane Domains = 22

Table 4: List of primers used in this study.

GENE	SEQUENCE (5' to 3')[a]	PRODUCT SIZE (bp)
PFB0435c	CAACTTTGCGTTCTCAGCAG (F)<br>TGTGAAGCCAACTGGAAAAA (R)	427
PFE0775c	CCGGACGTTTTGTATTAGATGG (F)<br>AAAGAAAGGAAGGAAGCGAGA (R)	400
PF11_0334	TGAGCCCAATACACTTGTTCA (F)<br>TCACCAGCGTGGTTATACTG (R)	458
PFB0210c	GAAGAATGGAAAGAGCGGATT (F)<br>ACACCATACGCACCCTTCTT (R)	453
PFI0955w	ATGTGCATAACGGCAACAAA (F)<br>TTCTCATATGCACCACCAAA (R)	437
PFI0785c	TGGAGTTTGGTTTGGTGGAT (F)<br>TCATGGTGAATCCAGCTTTT (R)	386
PFE1455w	ATGATTGGTGTGGGTGGAAT (F)<br>CAATAAGCTGCACGACATGG (R)	447
PFL0170w	GCCGCAAGTATGGAAGGTAA (F)<br>AAATCCAGGCCCTTCTCATT (R)	377
MAL6P1.133	CCAAAAAGAAATTGGAAGGG (F)<br>TTTGTGAATAAGGTCGGGAAA (R)	398
PFL0420w	TGTTTGGTTATACAATCGGTTTTG (F)<br>TTCATTTCATTTGCCCAACA (R)	430
PFL1515c	GGAATGTGTTCGTGTGTTCG (F)<br>AGATGAAATAGGGGAAGGGTCT (R)	497
MAL8P1.13	TGGCCATGTTTCAATCATTAG (F)<br>TTGGGAATGGTTTGGTAGGA (R)	473
PF10_0215	AGTTGGCCGCATTCACTAAT (F)<br>GCACAGGTTGTTAGGAATGAAA (R)	440
PF11_0172	CCATCGTTTATGGGGACATT (F)<br>TTTCGATGTGCTCCTCGTTT (R)	499
PFB0465c	GCCAGATTATATGCCCGAAA (F)<br>TCCCCAAAAGATCCTACCAA (R)	490
PFI1295c	GGGAACAATGTGACCTACCG (F)<br>TTTCTTTTGGACAGGAGAGACA (R)	396
PF13_0252	TGGTGTAGTGGCAGGATTAGC (F)<br>TTTTGTGTCCAACACCTGGA (R)	410
MAL8P1.32	CCGGTTGTTTGTCCAGAAAT (F)<br>TGTGGAACATCCATAGCCTG (R)	423
PFA0160c	GAAAAGCGAGGAGATCCAGA (F)<br>CCATCTTCGATAGAAAAATACGA (R)	471
PF14_0662	TGATTGTATCGATGGCTGATT (F)<br>TTTCATTTCATCATTCGGAAAA (R)	422
PFL1315w	AAAAAGAGAAGCGCATTGTTG (F)<br>TACCATCCGTTTGGGATTTC (R)	381
PF14_0622	TCCGAATTGTTTTGGGTGTT (F)<br>TTTCCTTGTGCTACTGATTCG (R)	400
PF14_0342	ATTAAGTTCCCAGCCGCTTT (F)<br>TGATTCTTTCTTCCGTATGTGC (R)	350
PFA0240w	TTCGGCCCTAAAATAACAGC (F)<br>ATGTGCCTCCATGGTAAAAA (R)	362
PFA0245w	TATACAGCGACCTCAGCGTG (F)<br>GCTGCAGCTCCTATAACGGT (R)	419
PFC0530w	TGTATTTGGGGAAACATTGAA (F)<br>CCTTGAGCTATTGCCTTTGG (R)	402
PFI0720w	TTTGGAATCTTGGGTTCAGC (F)<br>TTGTGGATGTGCCCATAAAG (R)	450
PF11_0310	TCAAACTGGGCTCAGTGGAT (F)<br>GATGCGTCGTCTCCATAGGT (R)	320
PF14_0679	ACGTTGGTGGTTTTGATCGT (F)<br>CACATTCAGCACCTCCCATA (R)	397
MAL13P1.206	GCAACGGTTTGGTTAGCTGT (F)<br>GAGCAGCACTCCAGTCTTCA (R)	406
PFB0275w	AGCGTTAGAAGCCGATTTGA (F)<br>AGGCAACGGTTGTTGTTACC (R)	374
PF11_0059	TGCTACCATGCTAGGACCATT (F)<br>AAAGAAACCTGAGCCCTTCC (R)	447
PF14_0260	CAGTTAGCTGGTTGTGTGCAA (F)<br>ACCACGGAAAACACGTAAGC (R)	480
PF14_0387	GCGAAGGAAATACAAAAGCA (F)<br>TGAAGCAAATGCTGAAGTGA (R)	401
β-Actin1	GTTGTTGACAACGGATCAGG (F)<br>CCAGTGGTACGACCAGAAGAA (R)	425

[a] Forward primer (F) and reverse primer (R).
